# Anatomy of the Epidemiological Literature on the 2003 SARS Outbreaks in Hong Kong and Toronto: A Time-Stratified Review

**DOI:** 10.1371/journal.pmed.1000272

**Published:** 2010-05-04

**Authors:** Weijia Xing, Gilles Hejblum, Gabriel M. Leung, Alain-Jacques Valleron

**Affiliations:** 1 INSERM, U707, Paris, France; 2 UPMC Université Paris 6, UMR S 707, Paris, France; 3 AP–HP, Hôpital Saint-Antoine, Unité de Santé Publique, Paris, France; 4 School of Public Health, Li Ka Shing Faculty of Medicine, The University of Hong Kong, Hong Kong; University of California San Francisco, United States of America

## Abstract

**Background:**

Outbreaks of emerging infectious diseases, especially those of a global nature, require rapid epidemiological analysis and information dissemination. The final products of those activities usually comprise internal memoranda and briefs within public health authorities and original research published in peer-reviewed journals. Using the 2003 severe acute respiratory syndrome (SARS) epidemic as an example, we conducted a comprehensive time-stratified review of the published literature to describe the different types of epidemiological outputs.

**Methods and Findings:**

We identified and analyzed all published articles on the epidemiology of the SARS outbreak in Hong Kong or Toronto. The analysis was stratified by study design, research domain, data collection, and analytical technique. We compared the SARS-case and matched-control non-SARS articles published according to the timeline of submission, acceptance, and publication. The impact factors of the publishing journals were examined according to the time of publication of SARS articles, and the numbers of citations received by SARS-case and matched-control articles submitted during and after the epidemic were compared. Descriptive, analytical, theoretical, and experimental epidemiology concerned, respectively, 54%, 30%, 11%, and 6% of the studies. Only 22% of the studies were submitted, 8% accepted, and 7% published during the epidemic. The submission-to-acceptance and acceptance-to-publication intervals of the SARS articles submitted during the epidemic period were significantly shorter than the corresponding intervals of matched-control non-SARS articles published in the same journal issues (*p*<0.001 and *p*<0.01, respectively). The differences of median submission-to-acceptance intervals and median acceptance-to-publication intervals between SARS articles and their corresponding control articles were 106.5 d (95% confidence interval [CI] 55.0–140.1) and 63.5 d (95% CI 18.0–94.1), respectively. The median numbers of citations of the SARS articles submitted during the epidemic and over the 2 y thereafter were 17 (interquartile range [IQR] 8.0–52.0) and 8 (IQR 3.2–21.8), respectively, significantly higher than the median numbers of control article citations (15, IQR 8.5–16.5, *p*<0.05, and 7, IQR 3.0–12.0, *p*<0.01, respectively).

**Conclusions:**

A majority of the epidemiological articles on SARS were submitted after the epidemic had ended, although the corresponding studies had relevance to public health authorities during the epidemic. To minimize the lag between research and the exigency of public health practice in the future, researchers should consider adopting common, predefined protocols and ready-to-use instruments to improve timeliness, and thus, relevance, in addition to standardizing comparability across studies. To facilitate information dissemination, journal managers should reengineer their fast-track channels, which should be adapted to the purpose of an emerging outbreak, taking into account the requirement of high standards of quality for scientific journals and competition with other online resources.

*Please see later in the article for the Editors' Summary*

## Abstract 2

Weijia Xing and colleagues reviewed the published epidemiological literature on SARS and show that less than a quarter of papers were published during the epidemic itself, suggesting that the research published lagged substantially behind the need for it.

## Editors' Summary

**Background:**

Every now and then, a new infectious disease appears in a human population or an old disease becomes much more common or more geographically widespread. Recently, several such “emerging infectious diseases” have become major public health problems. For example, HIV/AIDS, hepatitis C, and severe acute respiratory syndrome (SARS) have all emerged in the past three decades and spread rapidly round the world. When an outbreak (epidemic) of an emerging infectious disease occurs, epidemiologists (scientists who study the causes, distribution, and control of diseases in populations) swing into action, collecting and analyzing data on the new threat to human health. Epidemiological studies are rapidly launched to identify the causative agent of the new disease, to investigate how the disease spreads, to define diagnostic criteria for the disease, to evaluate potential treatments, and to devise ways to control the disease's spread. Public health officials then use the results of these studies to bring the epidemic under control.

**Why Was This Study Done?:**

Clearly, epidemics of emerging infectious diseases can only be controlled rapidly and effectively if the results of epidemiological studies are made widely available in a timely manner. Public health bulletins (for example, the *Morbidity and Mortality Weekly Report* from the US Centers from Disease Control and Prevention) are an important way of disseminating information as is the publication of original research in peer-reviewed academic journals. But how timely is this second dissemination route? Submission, peer-review, revision, re-review, acceptance, and publication of a piece of academic research can be a long process, the speed of which is affected by the responses of both authors and journals. In this study, the researchers analyze how the results of academic epidemiological research are submitted and published in journals during and after an emerging infectious disease epidemic using the 2003 SARS epidemic as an example. The first case of SARS was identified in Asia in February 2003 and rapidly spread around the world. 8,098 people became ill with SARS and 774 died before the epidemic was halted in July 2003.

**What Did the Researchers Do and Find?:**

The researchers identified more than 300 journal articles covering epidemiological research into the SARS outbreak in Hong Kong, China, and Toronto, Canada (two cities strongly affected by the epidemic) that were published online or in print between January 1, 2003 and July 31, 2007. The researchers' analysis of these articles shows that more than half them were descriptive epidemiological studies, investigations that focused on describing the distribution of SARS; a third were analytical epidemiological studies that tried to discover the cause of SARS. Overall, 22% of the journal articles were submitted for publication during the epidemic. Only 8% of the articles were accepted for publication and only 7% were actually published during the epidemic. The median (average) submission-to-acceptance and acceptance-to-publication intervals for SARS articles submitted during the epidemic were 55 and 77.5 days, respectively, much shorter intervals than those for non-SARS articles published in the same journal issues. After the epidemic was over, the submission-to-acceptance and acceptance-to-publication intervals for SARS articles was similar to that of non-SARS articles.

**What Do These Findings Mean?:**

These findings show that, although the academic response to the SARS epidemic was rapid, most articles on the epidemiology of SARS were published after the epidemic was over even though SARS was a major threat to public health. Possible reasons for this publication delay include the time taken by authors to prepare and undertake their studies, to write and submit their papers, and, possibly, their tendency to first submit their results to high profile journals. The time then taken by journals to review the studies, make decisions about publication, and complete the publication process might also have delayed matters. To minimize future delays in the publication of epidemiological research on emerging infectious diseases, epidemiologists could adopt common, predefined protocols and ready-to-use instruments, which would improve timeliness and ensure comparability across studies, suggest the researchers. Journals, in turn, could improve their fast-track procedures and could consider setting up online sections that could be activated when an emerging infectious disease outbreak occurred. Finally, journals could consider altering their review system to speed up the publication process provided the quality of the final published articles was not compromised.

**Additional Information:**

Please access these Web sites via the online version of this summary at http://dx.doi.org/10.1371/journal.pmed.1000272.

- The US National Institute of Allergy and Infectious Diseases provides information on emerging infectious diseases
- The US Centers for Control and Prevention of Diseases also provides information about emerging infectious diseases, including links to other resources, and information on SARS
- Wikipedia has a page on epidemiology (note that Wikipedia is a free online encyclopedia that anyone can edit; available in several languages)
- The World Health Organization has information on SARS (in several languages)

## Introduction

Emerging infectious diseases have become a major public health concern over the last two to three decades [1],[2]. When such an outbreak occurs, real-time collection, analysis, and dissemination of epidemiological information are key factors contributing to the effective and rapid control of the epidemic [3]. The first challenge for epidemiologists is to develop new surveillance and alert tools to detect in real time the events emerging anywhere in the world, and not just in highly developed countries [4],[5]. Upon detection of the outbreak, appropriate epidemiological studies should be launched immediately to help identify the causative agent, investigate the possible routes and modes of its transmission, define and validate diagnostic criteria, evaluate candidate treatments, forecast the spread of the epidemic, devise and evaluate evidence-based prevention, and monitor policies and strategies [6]–[8]. Hence, the outbreak of an emerging infectious disease causes a heavy epidemiological workload shared by public health specialists from national and international agencies, and academic epidemiologists. The importance of the rapid diffusion of public health information has long been recognized, and specialized international and national papers or Web bulletins are now made available during the course of outbreaks. However, journals remain the primary channel for communication of research. In this study, we analyzed the process according to which the results of academic epidemiological research are submitted and then formally published, during and after the outbreak of an emerging infectious disease. The proposed analysis concerns both the epidemiologists who submit their research to journals, and the journal editors who make decisions about the publication of the submitted research.

The 2003 severe acute respiratory syndrome (SARS) epidemic [9],[10] provided a model of an emerging infectious disease outbreak that occurred in the information-and-computerization age, which already has available real-time information systems, large databases, sophisticated statistical and mathematical analyses, and models. We studied the academic response to the SARS epidemic, and the resulting analysis may be viewed as a dissection of the scientific production on an epidemic—including the dynamics of this production in the course of the outbreak. Our analysis focused on four aspects: (1) distribution of the epidemiologists' workload among the different subspecialties and methodologies; (2) the characteristics of the epidemiological studies published in terms of design, data collection, statistical analysis, quality assurance, and quality control procedures used; (3) the timeline for the publication of the scientific results; and (4) the scientific impact of those publications as compared to control publications issued simultaneously.

## Materials and Methods

### Literature Search

We searched bibliographic databases for all published articles on epidemiology—defined as the study of the distribution and determinants of health-related states or events in specified populations, and the application of this study to control of health problems [11]—of the SARS epidemic in Hong Kong [12] and Toronto [13]. Hong Kong and Toronto were chosen because both cities, one in Asia and the other in North America, were strongly affected by the epidemic and have highly developed academic and public health infrastructures [14], and the results of the investigations performed on the outbreaks in these cities were almost exclusively published in English-language journals.

The entire literature identification–selection process is presented in Figure S1. Two literature searches were conducted: first, the MEDLINE database via PubMed and, second, the Science Citation Index Expanded and the Social Sciences Citation Index databases that we accessed via the Web of Science (Thomson Reuters). We searched for all journal articles written in English whose main studied subject was the 2003 SARS epidemic in Hong Kong and Toronto. Our study focused on articles published during the SARS epidemic and the 4 y thereafter (e.g., published between 1 January 2003 and 31 July 2007, assuming that most pertinent literature on an epidemic is produced within this period of time). We defined 5 July 2003, when the World Health Organization (WHO) reported that the last human chain of SARS transmission had been broken [15], as the end of the SARS epidemic.

The literature search was done in 2007, and updated on 11 February 2009. First, we used broad search equations to maximize retrieval sensitivity. Then, we finalized the search by removing all articles with at least one of the following seven exclusion criteria: (1) main study objective was not the study of SARS; (2) data analyzed in the study were not collected in Hong Kong or Toronto; (3) not a study on epidemiology (e.g., animal ecology); (4) not an original study (e.g., commentary, summary, review); (5) studies using only qualitative methodology, as defined by Critical Appraisal Skills Programme guidelines for qualitative research [16]; (6) study sample size ≤5; (7) full text of the article was unavailable: a request for a reprint of the article that we sent by e-mail to all corresponding authors of the articles for which we could not retrieve the full text through the Internet (either in open-access journals or in journals for which our institutions or the Inter-University Library of Medicine had a subscription) resulted in an absence of response. Two of the authors (WX and A-JV) independently applied the exclusion criteria to the same random sample of 100 articles. Good agreement was found between the two authors' selections (κ statistic: 0.86) [17]. Subsequently, review of the whole initial set of articles by the first author (WX) resulted in the final bibliographic database that was built with EndNoteX1 software (Thomson Reuters).

To compare the publication timelines of academic research articles with that of public health information, we also searched MEDLINE for public health reports mentioning the 2003 Hong Kong and/or Toronto SARS epidemic published in four public health bulletins during the SARS epidemic and the 4 y thereafter: *Morbidity and Mortality Weekly Report* for the Centers for Disease Control and Prevention (CDC), *The Weekly Epidemiological Record* and *Bulletin of the World Health Organization* for the WHO, and *Canada Communicable Disease Report* for the Public Health Agency of Canada. At the time of the SARS epidemic, *Communicable Diseases Watch*, a bulletin of the Centre for Health Protection, Department of Health of Hong Kong was not yet available (its first issue appeared on 13 June 2004). The literature search was done on 15 May 2009.

### Data Analyzed

Articles were classified into four large categories (investigation and surveillance, case management, prevention and control, psychobehavior) and in 11 research domains (Table 1) on the basis of the main objective of the study, as stated by the authors. We conducted a detailed analysis of the study designs, and the information given by the authors on the statistical, informatics, and quality-control methods. A data-collection grid was devised (Table S1). For each retained article, we recorded the study type and design, the type and size of the sample population, the software used for data management and statistical analyses, and the quality assurance and quality-control processes described in their Materials and Methods. The collected data were coded in a relational database (Access, Microsoft Office 2003).

**Table 1:** Breakdown of the 11 major epidemiological research domains according to categories 1–4.

Category	Research Domain	Detailed Research Objectives
**1. Investigation and surveillance**	Description of the outbreak	Time, place, and persons
	Search for causative agent	Identification
		Characteristics
	Transmission	Determine the modes and routes of transmission
		Estimate the transmission probability and variability
		Predict future trends of the present outbreak
	Risk factors	Determine the risk factors and disease determinants
**2. Case management**	Clinical presentations	Describe the clinical features, pathophysiological evolution, immune response, and associated clinical complications
	Diagnostic assays	Development and evaluation of their sensitivity and specificity
	Treatments and medical interventions	Assess efficacy and adverse events of treatment and medical interventions
	Prognosis	Describe patient outcomes
		Identify prognostic factors
	Medical decision making	Promote planning and policy-making by health services
**3. Prevention and control**	Prevention and control measures	Describe the use of specific control measures in the population
		Estimate and evaluate effectiveness of the control measures
		Develop methods and/or tools for real-time monitoring during the outbreak
**4. Psychobehavior**	Psychobehavioral investigations	Assess the level of personal knowledge and perception of risk
		Assess the personal attitudes and preventive measures taken regarding the outbreak threat
		Evaluate SARS-related individual psychological and social impacts

### Publication Timeline and Citations of SARS Studies

The timeline of publication process of the articles and their citations were determined for articles whose submission, acceptance, and publication dates were known. Submission and acceptance dates were obtained from the information provided by the journal. The publication date was defined as the date of the article's full text availability: when the date of print publication and the date of online publication were both available, the publication date was defined as the earliest of these. When only the month of publication was available, the publication date was set at the 15th of that month. The numbers of citations of articles were those provided by the Web of Science (Thomson Reuters) on 12–13 January 2009.

In order to find a potential particular pattern of the SARS studies in terms of publication timeline and citations, as compared to comparable studies in other fields, two case-control studies were performed. The first compared the submission-to-acceptance-to-publication sequence for SARS-case versus control articles. The control articles were defined as the two research articles following the SARS-case article published in the same volume and issue of the journal that did not concern SARS. The second case-control study compared the numbers of SARS-case and control article citations. In this analysis, the control articles of a case article were all other research articles published in the same issue and volume of the journal. The two case-control studies were based on the SARS articles submitted within 2 y after the epidemic, and analyses were done separately for articles submitted during and after the epidemic.

In addition, we studied the timeline trend of impact factors of the journals in which the SARS articles had been published. Those impact factors were obtained in the Journal Citation Reports database (Thomson Reuters).

### Statistical Analysis

The R statistical freeware (R version 2.6.1, The R Foundation for Statistical Computing) [18] software was used for obtaining all random samples and for all statistical analyses. The Kruskal-Wallis test was used to compare the medians of SARS articles' publication dates for the 11 research domains, and the submission-to-acceptance intervals, the acceptance-to-publication intervals, and the numbers of citations of SARS articles submitted during the epidemic and over the 2 y thereafter. The Kaplan-Meier method was used to represent the time-to-acceptance and the time-to-publication distributions, and then compared by the log-rank test. Cox models with a robust variance estimation that accounts for matching, were fitted to provide hazard ratio (HR) as a measure of the effect size, with 95% confidence interval (CI). The Wilcoxon signed-rank test was used to compare the distributions of the numbers of SARS article citations and of the citations of their corresponding control articles. Dispersions in individual values were expressed with interquartile range (IQR). The Jonckheere test [19] was used to assess the existence of a time trend for the median impact factor of journals in which SARS articles were published. In all tests, the statistical significance was defined as *p*≤0.05.

## Results

### Literature Search

The initial literature search provided 932 and 298 articles concerning the epidemic in Hong Kong and Toronto, respectively (see in Figure S1 the flowchart detailing the selection process of SARS articles). Applying the exclusion criteria resulted in the final selection of 263 and 58 articles for Hong Kong and Toronto, respectively. Because ten of the selected articles concerned the epidemic in both Hong Kong and Toronto, the final bibliographic database contained 311 different articles. Dates of online publication were obtained for 164 of the 311 SARS articles. Because ten articles had their online version posted after their print version, analyses involving publication dates were based on 154 online and 157 print publication dates. In addition, we selected a random sample of 100 out of the 263 articles dealing with the Hong Kong epidemic, and retained all articles dealing with the Toronto epidemic (*n* = 58) for further detailed analyses. These time-consuming analyses were performed on this subsample set of articles in order to facilitate feasibility. Because six studies concerned the epidemic in both cities, this final subset consisted of 152 articles. The detailed list of the whole set and subset articles (*n* = 311 and *n* = 152, respectively) is given in the Text S1.

The search of public health bulletins identified 29 SARS reports published on the Hong Kong and/or Toronto epidemic, among which 20 (69%) were published during the epidemic (see Figure S2): 12 in *The Weekly Epidemiological Record*, zero in *Bulletin of the World Health Organization*, two in *Morbidity and Mortality Weekly Report*, three in *Canada Communicable Disease Report*, and three others published first in *Morbidity and Mortality Weekly Report* and then in *Canada Communicable Disease Report*. These reports focused on the description of the outbreak situation (13/20), an update on the number of SARS cases (ten out of 20), prevention and control policies (ten out of 20), and descriptions of the clinical features or the case definition (seven out of 20). Such reports cannot obviously be considered as original peer-reviewed research articles and were not included in the studies related to the research domains or to citation impact.

### Typology of Studies

**Figure 1:**
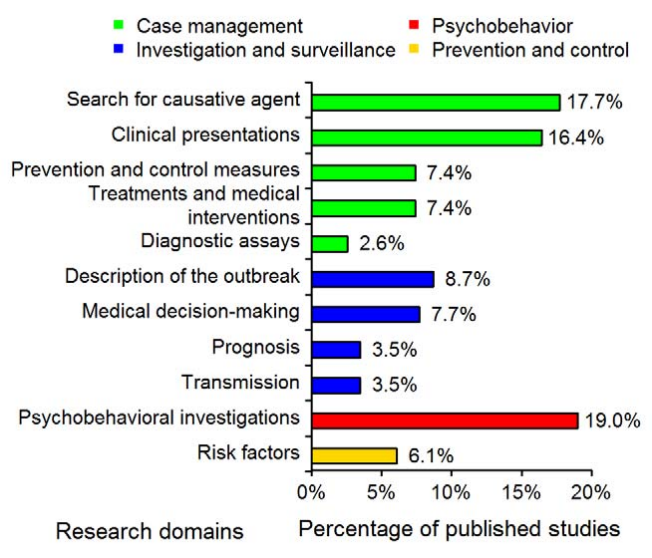
Distribution of the 311 SARS epidemiology papers in the 11 research domains (see Table 1). The studies corresponding to the “Case management” and “Investigation and surveillance” categories represented 52% and 23% of the 311 studies, respectively.

The distributions of the 11 research domains addressed by the 311 articles are shown in Figure 1. The majority (52%) of the published studies were in the “case-management” category, with 16% concerning the assessment of diagnostic tests or criteria. “Investigation and surveillance” represented 23% of the studies; 19% were “psychobehavioral studies”; and 6% concerned “prevention and control.”

The detailed analysis (Table 2) performed on the subset of 152 articles showed that the majority of the studies were descriptive epidemiology (54%), 42% were cohort studies, and 20% were cross-sectional studies. Case-control design and experimental studies were rare (5% and 6%, respectively). The majority of studies (70%) were conducted in hospitals. One-third of the studies used a questionnaire to collect data. Post mortem findings were reported in four articles. The use of data initially collected for another reason (hospital records, 13%) or secondary data analysis (i.e., the use of data already analyzed in previous studies, 32%) was frequent.

**Table 2:** Methodological characteristics of the published epidemiological studies on SARS.

Characteristic (*n* Articles)	Category	Subcategory	*n* (%^a^) Studies
**Type of study (152)**	Descriptive epidemiology		82 (54)
	Analytical epidemiology		45 (30)
	Theoretical epidemiology		16 (11)
	Experimental epidemiology		9 (6)
**Study design (152)**	Cross-sectional studies		30 (20)
	Cohort studies	Descriptive (longitudinal) cohort	32 (21)
		Prospective cohort	12 (8)
		Historical cohort	20 (13)
	Case-control studies		8 (5)
	Experimental studies	Intervention trials	2 (1)
		Clinical trials	7 (5)
	Mathematical modeling		16 (11)
	Molecular studies		11 (7)
	Diagnostic studies		14 (9)
**Case-definition (118)**	WHO and/or CDC case definition		80 (68)
	Local health authority's or authors' case definition		19 (16)
	Not specified		19 (16)
**Study setting (152)**	Hospital		106 (70)
	Community		33 (22)
	Hospital and community		13 (9)
**Data-collection instrument** b **(152)**	Questionnaire		50 (33)
	Biological specimen collection		60 (39)
	Physical examinations		33 (22)
	Environmental sample		2 (1)
	Hospital, medical, or exposure records		20 (13)
	Secondary data^c^		49 (32)
	Not specified		2 (1)
**Quality-assurance processes for data collection (152)**	Indicated in the article		53 (35)
	Not specified		99 (65)
**Quality-control activities for data collection (152)**	Indicated in the article		19 (13)
	Not specified		133 (88)
**Software for database management (152)**	Indicated in the article		8 (5)
	Not specified		144 (95)
**Software for data analysis (114)**	Indicated in the article		66 (58)
	Not specified		48 (42)

a Percentages do not add up to 100 because of rounding figures.

b Percentages in this section do not add up to 100% because multiple answers were possible.

c Reanalysis of previously used data.

### Study Population

**Figure 2:**
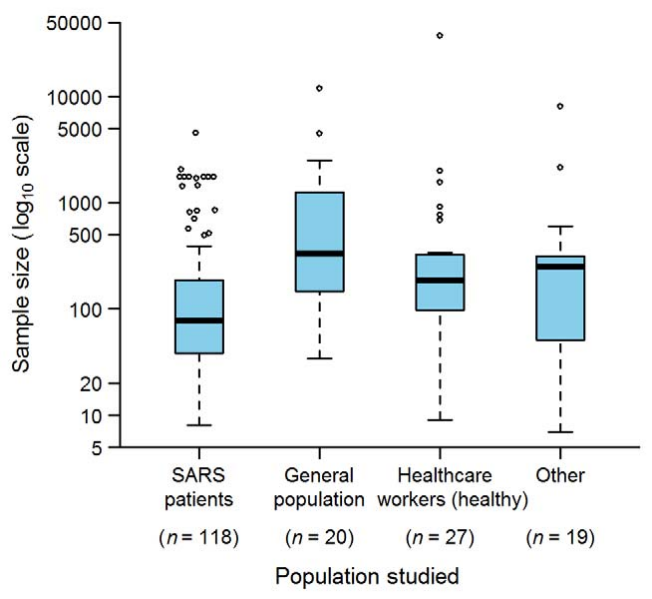
Sample sizes used in the epidemiological studies on SARS (152 articles). “Other” indicates studies in which patients without SARS, households of SARS patients, and quarantined individuals were studied. The sum of *n* is greater than 152 because several studies analyzed more than one population. Box-plot representation: The horizontal line inside the box represents the median; the lower and upper borders of the box represent the 25th and 75th percentiles, respectively; the whiskers correspond to extending to 1.5 times the box width (i.e., the IQR) from both ends of the box, and the circles represent values outside that interval. Whenever the minimum or maximum observed value is within the whisker interval, the alternative limit of the corresponding whisker becomes the corresponding minimum or maximum observed value.

The study population was composed of SARS patients in 118 (78%) studies, individuals from the general population in 20 (13%), non-SARS health care workers in 27 (18%), and other types (e.g., patients without SARS, households of SARS patients, and quarantined individuals) in 19 (12%) (Figure 2). The largest SARS-patient sample in a single study was 4,536 [20], obtained by pooling Hong Kong and Toronto patients with patients from other affected zones (e.g., Beijing, Singapore) to simulate strategies for controlling SARS outbreaks. The largest sample sizes for the other study population groups were: 12,000 for the general population [21] in a study estimating the seropositivity rate of the SARS coronavirus in the Hong Kong region; 37,174 for health care workers [22] in a study assessing the effectiveness of an herbal formula; and 8,044 for other populations [23] in a study estimating the ability of laboratory tests to discriminate SARS patients and patients with other causes of community-acquired pneumonia.

### Quality Assurance and Quality Control

Among the 118 studies including SARS patients, 16% did not specify the SARS case-definition criteria applied, 68% used the WHO and/or CDC criteria, and 16% used the criteria established by authors or local health authorities. Among the 120 (79%) studies that collected original data, only two specified double-data entries and ten stated that they checked the data for errors through manual or systematic inspection. For 70% of the 30 studies with data on chest radiographs, the radiologists' evaluations had been double-blinded. Researchers reported using statistical or mathematical techniques to analyze the data in 114/152 (75%) studies. Among them, statistical methods were not described in seven (6%) articles, and the software used to analyze the data was not indicated in 48 (42%). The database-management software was given in only 5% of the 152 studies.

### Journals Publishing SARS Articles

The 311 SARS articles comprising our entire database were published in 137 different journals. Among them, 50 (37%) journals published more than one SARS article. Three journals published more than ten articles: 32 (10%) publications appeared in *Emerging Infectious Diseases*, 11 (4%) in *The Lancet*, and 11 (4%) in *Radiology*. Seventeen studies were published during the epidemic. The first ten published studies appeared in *The Lancet* (7/10) and *The New England Journal of Medicine* (3/10). The next seven were published in *The Journal of the American Medical Association* (2/7), *Clinical Chemistry* (1/7), *Science* (1/7), *British Medical Journal* (2/7), and *Canadian Medical Association Journal* (1/7).

### Publication Timeline

**Figure 3:**
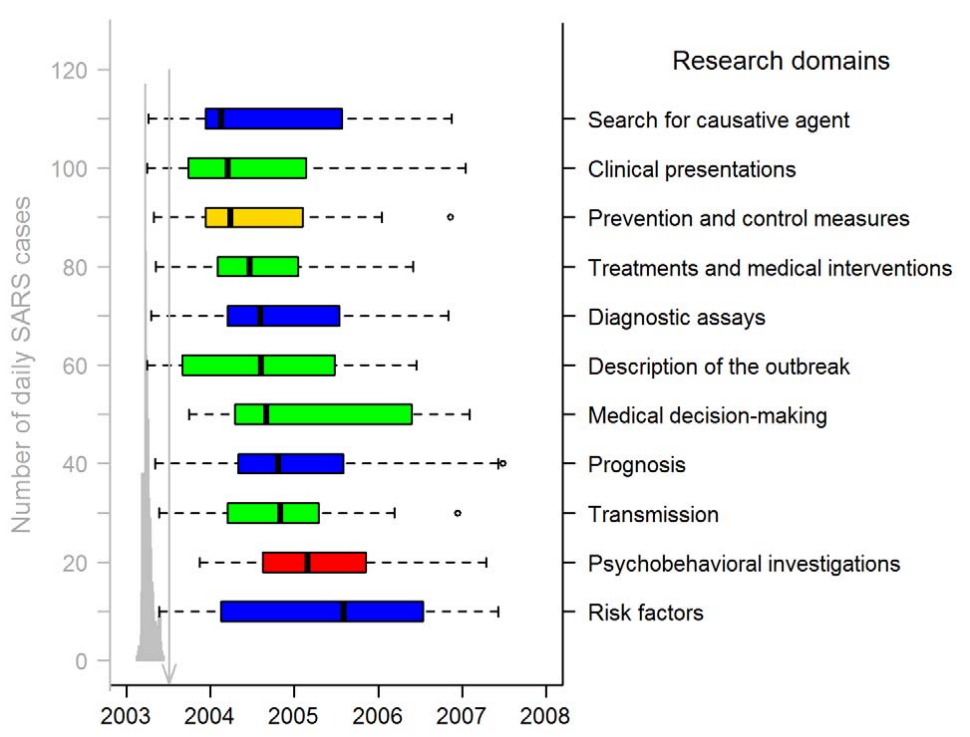
Publication dates of SARS papers in the 11 research domains (311 articles). The gray graph on the left shows the timing of the Hong Kong and Canadian epidemics (sum of both daily numbers of SARS cases), with a peak corresponding to 117 and 114 SARS cases on 24 and 25 March 2003. Hong Kong SARS data were from the SARSID database [33]; Canadian data were from the Public Health Agency of Canada [34]. The vertical line points to 5 July 2003, the date WHO declared that the last human chain of transmission had been broken. Box-plot representation: as in Figure 2 except box representation is horizontal; the colors indicate the study categories (see insert in Figure 1; green, case management; blue, investigation and surveillance; red, psychobehavior; yellow, prevention and control).

We derived the publication timeline for each of the research domains. Small percentages of articles were submitted or published for each research domain during the epidemic period. The maximum was 38% (21/55) of the articles in the category clinical presentations; the minimum was 3% (2/59) in the category psychobehavioral investigations. Only 10% (5/50), 17% (4/23), and 21% (4/19) of the articles dealing with diagnostic assays, treatments, and prevention and control, respectively, were submitted or published during the epidemic. The median publication dates for the different research domains (see Figure 3) differed significantly (*p*<0.001).

**Figure 4:**
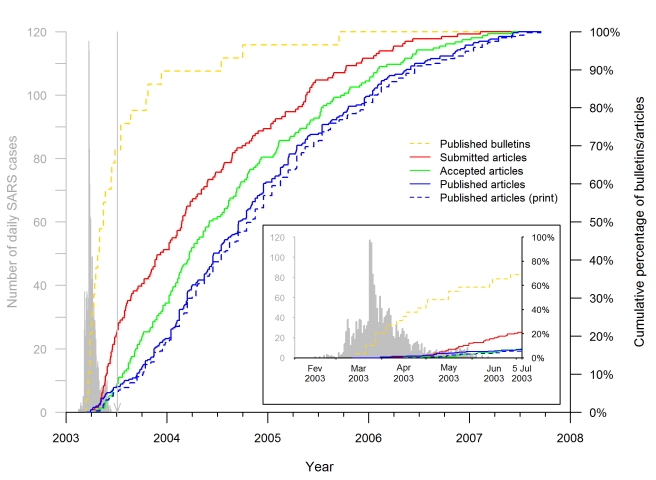
Timeline of SARS epidemiology publications on the Hong Kong and Toronto epidemic. The curves (red, green, and both blue lines) show the cumulative distributions of the 311 articles published by 15 September 2007, according to the publication and acceptance or submission dates for the 185 and 157 articles, respectively, for which the information was available. The solid blue line shows the cumulative distribution of the publication dates, defined as the earliest date of publication, print or online. The dotted blue line shows the cumulative distribution of the print publication dates. The dashed yellow line shows the cumulative distributions of the 29 public health bulletins published by 15 September 2005 according to their publication dates. For comparison, the timing of the Hong Kong and Canadian epidemic is shown in gray on the left, as described in the legend to Figure 3. The vertical line points to 5 July 2003, the date WHO declared that the last human chain of transmission had been broken. The insert is a superposition of the publication timeline and course of the epidemic.

The date-of-submission distribution indicates that the academic response to the epidemic was rapid (Figure 4). In Hong Kong and Toronto, respectively, the 2003 SARS epidemic started on 7 and 15 March, and lasted until 23 June and 2 July [15]. On 31 March, a few weeks after the onset of the epidemic, the first articles reporting on the Hong Kong [24] and Toronto [13] outbreaks were both published online in the same journal. However, only a minority of the total number of SARS articles submitted (34/157, 22%), accepted (14/185, 8%), or published (21/311, 7%, with four, one, and 16 articles published only in an online version, only in a print version, and in both versions, respectively) were available to the scientific community up to and including 5 July, the end of the epidemic. The median date of article submission was 27 February 2004 (IQR: 30 July 2003–15 January 2005); their median acceptance and publication dates were 30 June (IQR: 02 December 2003–02 June 2005) and 16 September 2004 (IQR: 15 February 2004–15 July 2005), respectively, 124 and 202 d later. The median date of article print publication was 31 October 2004 (IQR: 15 February 2004–15 August 2005), 48 d after the median date of the earliest publication.

**Figure 5:**
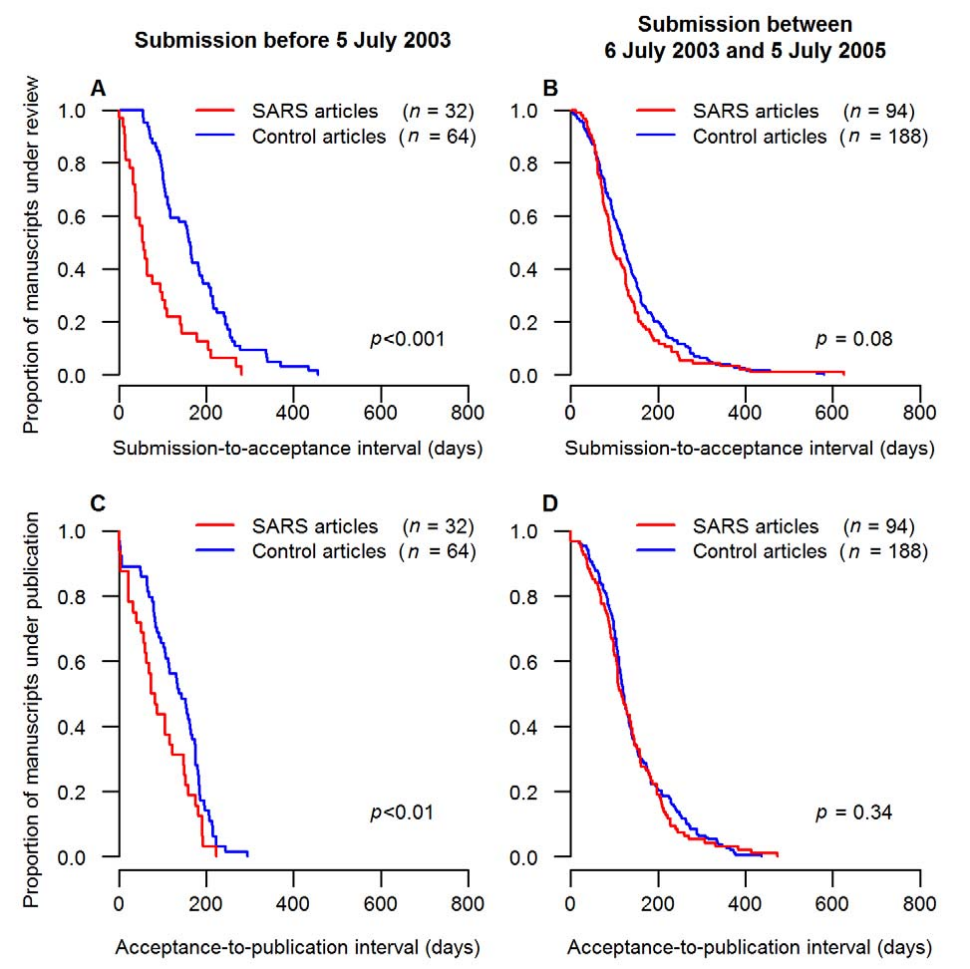
Comparison of publication intervals for case and control articles during and after the SARS epidemic. Submission, acceptance, and publication dates were available for 129 SARS articles submitted within 2 y (including 33 submitted during the epidemic), but were unavailable for three out of 129 couples of control. The Kaplan-Meier curves show the proportions of submitted manuscripts (ordinate) that took more than *x* d (abscissa) to be published. The comparisons of the submission-to-acceptance intervals between SARS articles and their control articles are shown for the SARS articles submitted (A) during the epidemic and (B) within 2 y after the end of the SARS epidemic. The comparisons of the acceptance-to-publication intervals between SARS articles and their control articles are shown for the SARS articles submitted (C) during the epidemic and (D) within 2 y after the end of the SARS epidemic.

Figure 5 shows the Kaplan-Meier curves representing the distributions of the submission-to-acceptance and acceptance-to-publication intervals for the SARS articles submitted during (left, Figure 5A and 5C, respectively) and after the epidemic (right, Figure 5B and 5D, respectively). The SARS articles submitted during the epidemic were accepted and published more rapidly than the non-SARS control articles (HR = 2.7, 95% CI 1.5–4.6, *p*<0.001 and HR = 1.7, 95% CI 1.1–2.5, *p*<0.01, respectively). The difference of median submission-to-acceptance intervals between SARS articles and their corresponding control articles was 106.5 d (95% CI 55.0–140.1) (Figure 5A); and the difference of median acceptance-to-publication intervals between SARS articles and their corresponding control articles was 63.5 d (95% CI 18.0–94.1) (Figure 5C). In contrast, the submission-to-acceptance and acceptance-to-publication intervals of the SARS articles submitted after the epidemic did not significantly differ from those of the control articles (*p* = 0.08 and *p* = 0.34, respectively). In addition, the submission-to-acceptance and acceptance-to-publication intervals for SARS articles submitted during the epidemic differed significantly from the corresponding intervals of SARS articles submitted over the 2 y thereafter (*p*<0.001 and *p*<0.01, respectively).

### Journal Impact Factors and Article Citations

**Figure 6:**
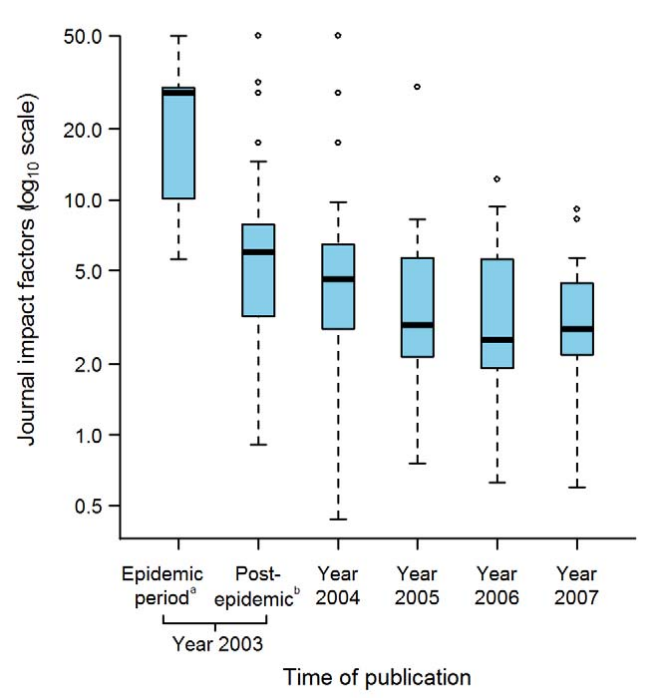
Journal Impact factors according to the time of publication of the 311 selected articles. The journal impact factors were obtained for 130 journals in which 299 SARS articles were published, the remaining 12 articles being published in seven journals that were not indexed in the Journal Citation Report database. ^a^Up to and including 5 July 2003, the date WHO declared that the last human chain of transmission had been broken. ^b^Between 6 July to 31 December 2003. Box-plot representation as described in Figure 2 legend: The horizontal line inside the box represents the median; the lower and upper borders of the box represent the 25th and 75th percentiles, respectively; the whiskers correspond to extending to 1.5 times the box width (i.e., the IQR) from both ends of the box, and the circles represent values outside that interval. Whenever the minimum or maximum observed value is within the whisker interval, the alternative limit of the corresponding whisker becomes the corresponding minimum or maximum observed value.

The impact factors were available for 130 journals in which 299 SARS articles were published; the remaining 12 articles were published in seven journals that were not indexed in the Journal Citation Report database. The impact factors of these 130 journals ranged between 0.437 and 50.017 (median of 4.131 [IQR: 2.51–6.50]). The impact factors of the journals in which the articles on SARS were published decreased significantly (*p*<0.0001) with publication dates (Figure 6). The case-control study on the citations received by the articles included 123 of the 129 case articles submitted within 2 y after the epidemic (33 during the epidemic, 90 after the epidemic) and 3,659 control articles, as the number of citations was not available for six of the 129 case articles submitted. The median numbers of citations of the SARS articles submitted during the epidemic and over the 2 y thereafter were 17 (IQR: 8.0–52.0) and eight (IQR: 3.2–21.8), respectively; this difference was significant (*p*<0.01). Considering these two periods, the number of SARS-article citations was significantly higher than the median number of citations of their control articles (15, IQR 8.5–16.5, *p*<0.05, and 7, IQR 3.0–12.0, *p*<0.01, respectively).

## Discussion

Herein we reported our analysis of the scientific literature on the epidemiology of the SARS epidemic in Hong Kong and Toronto taken as a model of an emerging infectious disease epidemic. We outlined the distribution of the workload among the traditional categories of epidemiological studies and methodologies. We showed that the time to disseminate study results could be quite long, in contrast to what would have been expected during a period of high public health alertness. The length of time to publish study results is dependent on several factors, which may classified as author-related (time to prepare the protocol, the questionnaires, to write the paper, and to choose the journal to submit to, and if rejected by this journal, to choose the next journals to submit to) or journal-related (time to find reviewers, to get reviews, to make a decision for publication, to publish). Our results indicate that the SARS articles submitted during the epidemic that were eventually published were more rapidly processed than control articles.

We chose to analyse a subset of the studies made on SARS, and to restrict our analysis to data that might be accurately quantified; in particular we only analyzed published papers. Hong Kong and Toronto accounted for only 2,005 (24%) of SARS cases out of a total of 8,422 cases worldwide. Nevertheless, Hong Kong and Toronto are indeed the cities with the highest number of cases in their respective continents, Asia and America. We also refrained from attempting to obtain qualitative data on each paper, as it could only have been done through surveying authors, editors, and journal managers to learn the numbers of previous submission of their papers (if any) by the authors, the nature of the decision process by editorial boards, etc. This information might be of great interest, but would result in low quality data, exhaustivity, and bias being highly dependent on the qualitative data on the papers. Therefore, we chose to analyse limited information, available in public databases, and set up a design allowing an unbiased comparison of SARS and control articles, and replication of the results.

Our detailed results showed that, during the outbreak of an emerging infectious disease, descriptive epidemiology predominated in the published literature (Table 2). Nearly four-fifths (78%, see Results, “Population Study”) of the published articles concerned studies on SARS patients conducted in a clinical setting. A large number of these investigations were devoted to assessing real-time diagnostic tools. At the same time, 22% of the retrieved articles did not concern SARS patients per se, reflecting the broader need of investigation in the community at-large when mitigation strategies are addressed. In particular, the results of our analysis highlighted the high proportion of published articles on psychobehavioral investigations (Figure 1): during such an outbreak, these investigations are indeed key elements for the implementation of control policies in the hospital and in the general population.

In large measure, the identified articles had relevance for public health authorities during the epidemic. However, only very few of such articles were submitted or published during the SARS epidemic (see Figure 4 and Results, “Publication Timeline”). Some public health information of immediate interest to the general public and health authorities and personnel were provided in a timely manner through specialized international or national public health bulletins: the first report published on the SARS epidemic mentioning Hong Kong and Vietnam was published on 14 March 2003 [25], 2 d after the first WHO alert concerning the SARS threat, and 69% of the public health information was published before the end of the SARS epidemic (Figure 4). However, our analysis of peer-reviewed journal articles (the main target of our study) quantified a worrying fact. Although academic articles providing data on this emerging disease received good impact scores in terms of the numbers of citations, they took a long time to be accepted and published (Figure 5), even though our findings demonstrated that journal managers had already accelerated the publication process during the epidemic (Figure 5A and 5C). The interval between submission and publication, and especially the acceptance-to-publication interval, is an important issue since many academic articles were aimed at solving practical clinical and epidemiological problems. The task of journal managers is difficult because speed to publication, although necessary, should not compromise review or the standard of quality of accepted articles. Our findings might encourage even more scientific journal managers to widely open online sections that could be activated upon detection of an emerging infectious disease outbreak, and possibly use a different review-publication system, so as to enhance the speed of publication. For example, several journals propose a specific section of manuscript submission involving dedicated fast management of those manuscripts that deserve rapid evaluation (e.g., “fast-track publication” in *The Lancet*, “rapid review” in *The New England Journal of Medicine*).

Visible changes in the dissemination of scientific information do not only concern speed of publication, they also acknowledge the technological and societal changes in the use of modern communication tools (Web 2.0). In 2007, the *Nature Publishing Group* launched *Nature Precedings*, which aims to rapidly disseminate, share, and discuss prepublication data [26]. More recently, the *BMJ* group launched a Web site on the 2009 H1N1/A pandemic flu [27] where articles, podcasts, learning modules, and other resources are available on a range of issues. *PLoS Currents: Influenza* is another such recent initiative [28] for rapid and open sharing of new scientific data, analyses, and ideas, the submissions being rapidly screened by a group of moderators who are leading researchers in the field. The rapid progress of information systems is likely to facilitate further similar initiatives. The methodology and the results of our study may be used in the future as a basis for estimating the impact of such changes in the publishing landscape in terms of speed of dissemination and quality.

Our results indicate that the publication delay is also heavily dependent on the researchers themselves: 78% of the articles analyzed in this study (i.e., only those eventually published) were submitted after the epidemic. In some instances, this delay could be explained by a previous unsuccessful submission to a high impact journal during the epidemic before the final submission, which is the only one that could be analyzed herein. However, the primary cause of the delay is undoubtedly the time necessary for the authors to design the study, acquire the data, and finalize the paper. This bottleneck could be reduced by developing a series of ready-to-use information technologies, to improve timeliness and, thus relevance, and further, to improve standardization, and thus comparability across studies in the event of an outbreak. Just as theoretical modelers have shifted to real-time approaches, for example, to estimate the basic/effective reproduction number of an epidemic [29], “field epidemiologists” should benefit from real-time tools for protocol writing and questionnaire design, and have them readily available on the Web so as to be prepared for the next emerging disease outbreak. Others, too, have advocated the need to prepare ready-to-use forms for clinical research on the 2009 H1N1/A pandemic [30]. Specific information systems dedicated to real-time research on the epidemic would provide valuable assistance for designers of future studies. Inspired by the guidelines recommended by STROBE [31] for observational studies, and CONSORT [32] for randomized controlled trials, such systems would offer standardized tools and protocols to assure adherence to good epidemiological practices. Our observations strongly suggest the need for coordinated international action dedicated to the development of such systems that, surprisingly, are not yet available in our information age.

The SARS outbreaks in Hong Kong and Toronto occurred nearly 7 y ago; and this amount of time was required to study the timeline of publications on these outbreaks, especially for analyzing citations of the SARS papers with an appropriate perspective. The approach we present here, which chronicles the academic response to an outbreak, will be useful to assess the changes in information dissemination that are underway.

## Supporting Information

Figure S1 Literature-selection procedure for studies on the SARS epidemic in peer-reviewed journals.
(0.12 MB PDF)

Figure S2 Timeline of publication of the reports issued in the weekly bulletins of WHO, CDC, or Health Canada during the epidemic.
(0.17 MB PDF)

Table S1 Data-collection grid to extract information from each selected article.
(0.13 MB DOC)

Text S1 List of the 311 selected article references.
(1.01 MB DOC)

## Abbreviations

CDC: Centers for Disease Control and Prevention
CI: 95% confidence interval
HR: hazard ratio
IQR: interquartile range
SARS: severe acute respiratory syndrome
WHO: World Health Organization
