# Supplementary material for: Anatomy of the Epidemiological Literature on the 2003 SARS Outbreaks in Hong Kong and Toronto: A Time-Stratified Review
Source: PLoS Med. 2010 May 4;7(5):e1000272. doi: 10.1371/journal.pmed.1000272 (PMC2864302; doi:10.1371/journal.pmed.1000272)
Supplement: Figure S2 — Timeline of publication of the reports issued in the weekly bulletins of WHO, CDC, or Health Canada during the epidemic. (0.17 MB PDF) [file pmed.1000272.s002.pdf]

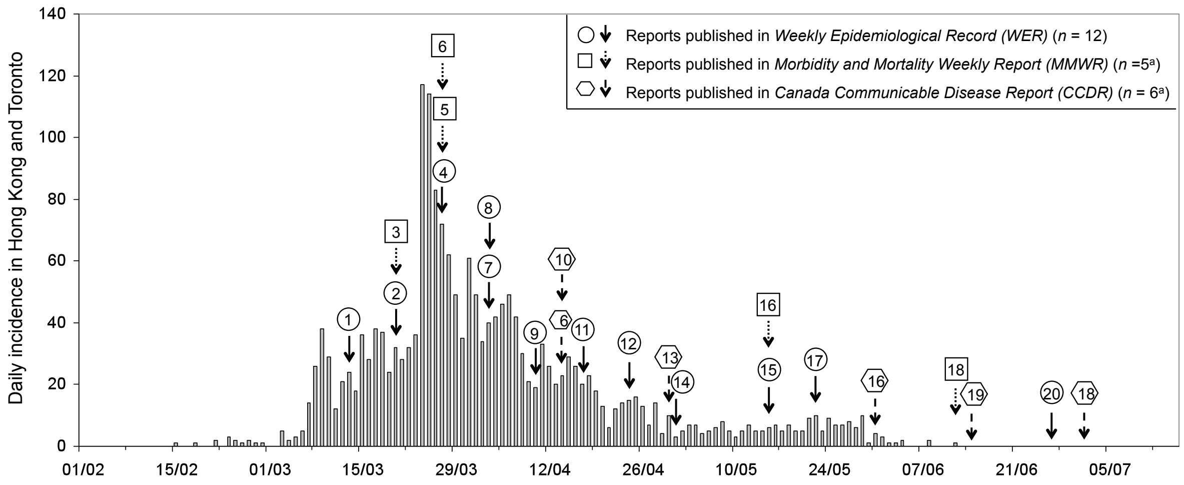

Figure S2. Timeline of the publication of the reports issued the weekly bulletins of WHO, CDC, or Health Canada during the epidemic.

[a]The 5 and 6 reports respectively published in MMWR and in CCDR include 3 reports published in both bulletins (items n° 6, 16 and 18 are shown twice). During the SARS epidemic (i.e., up to July 05, 2003), 20 of 29 bulletins/reports were published in *The Weekly Epidemiological Record*, *Morbidity and Mortality Weekly Report*, and *Canada Communicable Disease Report*. These reports summarize the various recommendations, the general information of the SARS epidemic (e.g., epidemiology, clinical features, diagnosis, laboratory, treatment, measures of infection control), and the situation of the epidemic in Hong Kong or Toronto. The timeline of their publication date and a brief summary of each report are shown in the Figure. Each figure associated with an arrow in the Figure corresponds to a report. The principal information in each report was the following:

1 14/03/2003 (publication date): WHO announced the outbreaks of a severe form of pneumonia in Guangdong, Hong Kong and Vietnam and describes the associated outbreak situation in these regions [1].

2 21/03/2003: a) update on the number of SARS cases - worldwide; b) case definition criteria; c) preliminary description of the epidemiology, the clinical presentation, the treatment and the laboratory findings of SARS [2].

3 21/03/2003: CDC described the scope of the outbreak, preliminary case definition, and interim infection control guidance for the United States [3].

4 28/03/2003: a) WHO's Global Outbreak Alert and Response Network coordinated an international multicentre research project to expedite identification of the causative agent, and to improve

diagnostic precision; b) the first global "grand rounds" on clinical features and treatment of SARS were held by WHO on March 26 ; c) update on the number of SARS cases – worldwide [4].

5 28/03/2003: a) description of an unusual cluster of cases associated with a hotel in Hong Kong (contact tracing and chain of the transmission in the cluster); b) identification of the potential etiologic agent of SARS [5].

6 28/03/2003, 15/04/2003: summaries of the clinical description of patients with SARS based on information collected since mid-February 2003 in Hong Kong, Taiwan, Bangkok, Singapore, the United Kingdom, Slovenia, Canada, and the United States [6,7].

7 04/04/2003: WHO issued recommendations for the screening of air passengers leaving affected areas and for the traveler safety to Hong Kong and Guangdong province of China [8].

8 04/04/2003: a) revision of the SARS case definitions; b) information for case reporting [9].

9 11/04/2003: description of the collaborative work of SARS diagnosis and the current available diagnostic tests [10].

10 15/04/2003: summary of the epidemiology, the clinical presentation and the laboratory findings based on the first 11 cases of SARS identified in Canada [11].

11 18/04/2003: a) description of the outbreak situation in China and of the scientific and clinical knowledge on SARS; b) update on the number of cases and on affected countries [12].

12 25/04/2003: WHO extended its SARS-related travel advice to Beijing and Shanxi province of China and to Toronto, Canada [13].

13 01/05/2003: a) summary of the current epidemiology of SARS in Canada; b) description of the actions that have taken place to date to manage the emerging situation in Canada [14].

14 02/05/2003: WHO lifted its SARS-related travel advice to Toronto, Canada [15].

15 16/05/2003: a) WHO removed Toronto from the list of areas with recent local transmission and from the list of travel advice areas; b) description of the current situation in Singapore and Hong Kong; c) WHO issued recommendations for the measures of infection control to break the chain of transmission from infected to healthy persons [16].

16 16/05/2003, 01/06/2003: summary of a cluster of SARS cases among healthcare workers in a hospital of Toronto that occurred despite apparent compliance with recommended infection-control precautions [17,18].

17 23/05/2003: a) report on Guangxi province of China visit; b) description of the situation in Taiwan; c) description of the risk of SARS transmission during air travel; d) update on the number of cases and on the affected countries [19].

18 13/06/2003, 01/07/2003: description of a second wave of SARS cases among patients, visitors, and healthcare workers that occurred at a Toronto hospital [20,21].

19 15/06/2003: overview of the surveillance and policy measures that Health Canada has implemented to address this concern as well as the initial results of passenger contact tracing [22].

20 27/06/2003: overview and commentary of WHO: over 100 days into the outbreak of SARS [23].

References

1. (2003) Acute respiratory syndrome. China, Hong Kong Special Administrative Region of China, and Viet Nam. Wkly Epidemiol Rec 78: 73-74.
2. (2003) Severe acute respiratory syndrome (SARS). Wkly Epidemiol Rec 78: 81-83.
3. (2003) Outbreak of severe acute respiratory syndrome--worldwide, 2003. MMWR Morb Mortal Wkly Rep 52: 226-228.
4. (2003) Severe acute respiratory syndrome (SARS). Wkly Epidemiol Rec 78: 89.
5. (2003) Update: Outbreak of severe acute respiratory syndrome--worldwide, 2003. MMWR Morb Mortal Wkly Rep 52: 241-246, 248.
6. (2003) Preliminary clinical description of severe acute respiratory syndrome. MMWR Morb Mortal Wkly Rep 52: 255-256.
7. (2003) Preliminary clinical description of severe acute respiratory syndrome. Can Commun Dis Rep 29: 69-70.
8. (2003) WHO recommended measures for persons undertaking international travel from areas affected by severe acute respiratory syndrome (SARS). Wkly Epidemiol Rec 78: 97-99.
9. (2003) Global surveillance for severe acute respiratory syndrome (SARS). Wkly Epidemiol Rec 78: 100-119.
10. (2003) WHO Multicentre Collaborative Networks for Severe Acute Respiratory Syndrome (SARS) diagnosis. Wkly Epidemiol Rec 78: 121-122.
11. (2003) Epidemiology, clinical presentation and laboratory investigation of severe acute respiratory syndrome (SARS) in Canada, March 2003. Can Commun Dis Rep 29: 71-75.

12. (2003) Severe Acute Respiratory Syndrome (SARS) in China and status of scientific and clinical knowledge, 14 April 2003. Wkly Epidemiol Rec 78: 129-130.

13. (2003) WHO extends its SARS-related travel advice to Beijing and Shanxi Province (China) and to Toronto (Canada), 23 April 2003. Wkly Epidemiol Rec 78: 137-138.

14. (2003) The war against an unknown pathogen: rising to the SARS challenge. Can Commun Dis Rep 29: 77-82.

15. (2003) Travel advice for Toronto (Canada) lifted. Wkly Epidemiol Rec 78: 145-146.

16. (2003) Severe acute respiratory syndrome. Wkly Epidemiol Rec 78: 169-170.

17. (2003) Cluster of severe acute respiratory syndrome cases among protected health-care workers--Toronto, Canada, April 2003. MMWR Morb Mortal Wkly Rep 52: 433-436.

18. Ofner M, Lem M, Sarwal S, Vearncombe M, Simor A (2003) Cluster of severe acute respiratory syndrome cases among protected health care workers-Toronto, April 2003. Can Commun Dis Rep 29: 93-97.

19. (2003) Severe acute respiratory syndrome (SARS). Wkly Epidemiol Rec 78: 181-183.

20. (2003) Update: severe acute respiratory syndrome--Toronto, Canada, 2003. MMWR Morb Mortal Wkly Rep 52: 547-550.

21. Wallington T, Berger L, Henry B, Shahin R, Yaffe B, et al. (2003) Update: severe acute respiratory syndrome--Toronto, 2003. Can Commun Dis Rep 29: 113-117.

22. Flint J, Burton S, Macey JF, Deeks SL, Tam TW, et al. (2003) Assessment of in-flight transmission of SARS--results of contact tracing, Canada. Can Commun Dis Rep 29: 105-110.

23. (2003) Severe acute respiratory syndrome (SARS): over 100 days into the outbreak. Wkly Epidemiol Rec 78: 217-220.
