# Supplementary material for: Anatomy of the Epidemiological Literature on the 2003 SARS Outbreaks in Hong Kong and Toronto: A Time-Stratified Review
Source: PLoS Med. 2010 May 4;7(5):e1000272. doi: 10.1371/journal.pmed.1000272 (PMC2864302; doi:10.1371/journal.pmed.1000272)
Supplement: Table S1 — Data-collection grid to extract information from each selected article. (0.13 MB DOC) [file pmed.1000272.s003.doc]

**Table S1. Data-collection grid to extract information from each article**

| **Section** | **Variable Name** | **Information** | **Format Specification** | **Comment** |
| --- | --- | --- | --- | --- |
| **Article reference** | ID | Unique index specific of the article | Incremental integer automatically generated by the database system | e.g., 1, 2, 3, …, 311 |
|  | Source | From which study subset | Dropdown menu list: |  |
|  |  |  | Hong Kong subset |  |
|  |  |  | Toronto subset |  |
|  |  |  | Combined subset |  |
|  | Authors | Full names and initials | Text | e.g., Lee, T. S. |
|  | Title | Title of the article | Text | Full title |
|  | Journal | Title of the journal | Journal full or abbreviated name according to either the Medline or Web of Science format | Medline format (e.g., Emerg Infect Dis); Web of Science format (e.g., Emerging Infectious Diseases) |
|  | Year | Publication year of the article | Number |  |
|  | Volume | Volume in which the article was published | Number |  |
|  | Issue | Issue in which the article was published | Text |  |
|  | Pages | The article’s first and last page numbers in the journal | Text | e.g., 352–58 |
|  | r_date | Date of reception of the first manuscript submission | Date format: dd/mm/yyyy | Only entered when mentioned in the article or the Medline database |
|  | a_date | Date of manuscript acceptance | Date format: dd/mm/yyyy | Only entered when mentioned in the article or the Medline database |
|  | e_date | Date of the online publication | Date format: dd/mm/yyyy | Only entered when mentioned in the article or the Medline database |
|  | p_date | Date of the print publication | Date format: dd/mm/yyyy | Only entered when mentioned in the article or in the Medline database |

Table continues on the following page.

**Table S1.** (Continued)

| **Section** | **Variable Name** | **Information** | **Format Specification** | **Comment** |
| --- | --- | --- | --- | --- |
| **Classification of the study** | Type | Study type | Dropdown menu list: |  |
|  |  |  | Descriptive epidemiology |  |
|  |  |  | Analytic epidemiology |  |
|  |  |  | Theoretical epidemiology |  |
|  |  |  | Experimental epidemiology |  |
|  | Domain | Main research domain of the study | Dropdown menu list: |  |
|  |  |  | Description of the outbreak |  |
|  |  |  | Search for causative agent |  |
|  |  |  | Transmission |  |
|  |  |  | Risk factors |  |
|  |  |  | Clinical presentations |  |
|  |  |  | Diagnostic assays |  |
|  |  |  | Treatments and medical interventions |  |
|  |  |  | Prognosis |  |
|  |  |  | Medical decision-making |  |
|  |  |  | Prevention and control measures |  |
|  |  |  | Psychobehavioral investigation |  |
|  | Setting | Study setting | Dropdown menu list: | Hospital study refers to those conducted in any medical setting (e.g., hospital, clinic) |
|  |  |  | Hospital |  |
|  |  |  | Community |  |
|  |  |  | Both |  |
|  | Design | Epidemiological study design | Dropdown menu list: |  |
|  |  |  | Cross-sectional study |  |
|  |  |  | Descriptive cohort study (longitudinal study) |  |
|  |  |  | Prospective cohort study |  |
|  |  |  | Historical cohort study |  |
|  |  |  | Case–control study |  |
|  |  |  | Intervention trial |  |
|  |  |  | Clinical trial |  |
|  |  |  | Mathematical modeling study |  |
|  |  |  | Molecular study |  |
|  |  |  | Diagnostic study |  |

Table continues on the following page.

**Table S1.** (Continued)

| **Section** | **Variable Name** | **Information** | **Format Specification** | **Comment** |
| --- | --- | --- | --- | --- |
| **Characteristics of the study population** | Population | Type of population | Check boxes: |  |
|  |  |  | SARS patients |  |
|  |  |  | General population |  |
|  |  |  | Healthcare workers (non SARS) |  |
|  |  |  | Other^a^ |  |
|  | Definition | Definition criteria of the SARS cases | Check boxes: |  |
|  |  |  | WHO SARS-case definition |  |
|  |  |  | CDC SARS-case definition |  |
|  |  |  | Other^a^ |  |
|  |  |  | Not specified |  |
|  | Location | Population recruitment location | Check boxes: |  |
|  |  |  | Hospital |  |
|  |  |  | Place of work |  |
|  |  |  | Place of residence |  |
|  |  |  | Other^a^ |  |
|  |  |  | Not specified |  |
|  | Sample_size | Sample size of the study | Number |  |
|  | ss_init | Initial sample size of the study | Number | Only entered when applicable, depending on study design |
|  | ss_fin | Final sample size of the study | Number | Only entered when applicable, depending on study design |
|  | data_coll | Type of data collection | Check boxes: | Secondary data refer to analyses of data that had been collected and used in a previous study. |
|  |  |  | Questionnaire |  |
|  |  |  | Biological specimen collection |  |
|  |  |  | Physical examination(s) |  |
|  |  |  | Environmental sample |  |
|  |  |  | Hospital, medical or exposure records |  |
|  |  |  | Secondary data |  |
|  |  |  | Other^a^ |  |

Table continues on the following page.

**Table S1.** (Continued)

| **Section** | **Variable Name** | **Information** | **Format Specification** | **Comment** |
| --- | --- | --- | --- | --- |
| **Characteristics of the study data** | dc_start | First date of data collection | Date format: dd/mm/yyyy |  |
|  | dc_end | Last date of data collection | Date format: dd/mm/yyyy |  |
|  | Quality | Procedures used to guarantee the quality of data | Text | e.g., double data entry, data checking procedure(s) |
|  | Analysis | Description of the statistical or mathematical methods used in the study | Yes**^b^**/not specified/not applicable |  |
|  | sft_dm | Software used for data management | Yes**^b^**/not specified |  |
|  | sft_da | Software used for data analysis | Yes**^b^**/not specified/not applicable |  |

^a^In such a case, a dependent variable is entered (text format) to specify the meaning of “other”.

^b^In such a case, a dependent variable is entered through an incremental group of checked boxes to indicate the method(s) or the software used.
