# Supplementary material for: Anatomy of the Epidemiological Literature on the 2003 SARS Outbreaks in Hong Kong and Toronto: A Time-Stratified Review
Source: PLoS Med. 2010 May 4;7(5):e1000272. doi: 10.1371/journal.pmed.1000272 (PMC2864302; doi:10.1371/journal.pmed.1000272)
Supplement: Text S1 — List of the 311 selected article references. (1.01 MB DOC) [file pmed.1000272.s004.doc]

**Text S1. List of the 311 selected-article references** (listed in alphabetical order by group)

Hong Kong subset (253 studies): [1–253]

Toronto subset (48 studies): [254–301]

Hong Kong and Toronto combined subset (10 studies): [302–311]

Hong Kong subsample (94 studies): [4,6,8,12,15,16,18–21,32,34,44,48,49,51,55,57,58,61,62,68,73,80–83,87,91–93,96–100,103,107,109,111,114,119,120,124–126,131,132,135,139,142–146,157,159,160,163,164,168,172,173,175–177,182,184,187,191,193,194,196,198,202,204,205,207,214,217,219,222,224,232–234,236,239–241,243,246,250,251]

Hong Kong and Toronto subsample (6 studies): [302,306,307,309–311]

1. Antonio GE, Ooi CG, Wong KT, Tsui EL, Wong JS, et al. (2005) Radiographic-clinical correlation in severe acute respiratory syndrome: study of 1373 patients in Hong Kong. Radiology 237: 1081-1090.

2. Antonio GE, Wong KT, Hui DS, Wu A, Lee N, et al. (2003) Thin-section CT in patients with severe acute respiratory syndrome following hospital discharge: preliminary experience. Radiology 228: 810-815.

3. Antonio GE, Wong KT, Tsui EL, Chan DP, Hui DS, et al. (2005) Chest radiograph scores as potential prognostic indicators in severe acute respiratory syndrome (SARS). AJR Am J Roentgenol 184: 734-741.

4. Auyeung TW, Lee JS, Lai WK, Choi CH, Lee HK, et al. (2005) The use of corticosteroid as treatment in SARS was associated with adverse outcomes: a retrospective cohort study. J Infect 51: 98-102.

5. Bradford IM (2004) Tales from the frontline: the colorectal battle against SARS. Colorectal Dis 6: 121-123.

6. Cauchemez S, Boelle PY, Donnelly CA, Ferguson NM, Thomas G, et al. (2006) Real-time estimates in early detection of SARS. Emerg Infect Dis 12: 110-113.

7. Caves ND, Irwin MG (2006) Attitudes to basic life support among medical students following the 2003 SARS outbreak in Hong Kong. Resuscitation 68: 93-100.

8. Chan BC, Lee CP, Tang GW (2004) Universal SARS preventive measures in an obstetrics unit: experience of health care staff. Am J Infect Control 32: 417-420.

9. Chan HL, Kwan AC, To KF, Lai ST, Chan PK, et al. (2005) Clinical significance of hepatic derangement in severe acute respiratory syndrome. World J Gastroenterol 11: 2148-2153.

10. Chan JC, Tsui EL, Wong VC (2007) Prognostication in severe acute respiratory syndrome: a retrospective time-course analysis of 1312 laboratory-confirmed patients in Hong Kong. Respirology 12: 531-542.

11. Chan JW, Ng CK, Chan YH, Mok TY, Lee S, et al. (2003) Short term outcome and risk factors for adverse clinical outcomes in adults with severe acute respiratory syndrome (SARS). Thorax 58: 686-689.

12. Chan KC, Tang NL, Hui DS, Chung GT, Wu AK, et al. (2005) Absence of association between angiotensin converting enzyme polymorphism and development of adult respiratory distress syndrome in patients with severe acute respiratory syndrome: a case control study. BMC Infect Dis 5: 26.

13. Chan KH, Cheng VC, Woo PC, Lau SK, Poon LL, et al. (2005) Serological responses in patients with severe acute respiratory syndrome coronavirus infection and cross-reactivity with human coronaviruses 229E, OC43, and NL63. Clin Diagn Lab Immunol 12: 1317-1321.

14. Chan KH, Poon LL, Cheng VC, Guan Y, Hung IF, et al. (2004) Detection of SARS coronavirus in patients with suspected SARS. Emerg Infect Dis 10: 294-299.

15. Chan KS, Lai ST, Chu CM, Tsui E, Tam CY, et al. (2003) Treatment of severe acute respiratory syndrome with lopinavir/ritonavir: a multicentre retrospective matched cohort study. Hong Kong Med J 9: 399-406.

16. Chan KY, Ching JC, Xu MS, Cheung AN, Yip SP, et al. (2007) Association of ICAM3 genetic variant with severe acute respiratory syndrome. J Infect Dis 196: 271-280.

17. Chan LS, Cheung GT, Lauder IJ, Kumana CR (2004) Screening for fever by remote-sensing infrared thermographic camera. J Travel Med 11: 273-279.

18. Chan LY, Wong JT, Li PK, Lui SF, Fung H, et al. (2004) Risk of transmission of severe acute respiratory syndrome to household contacts by infected health care workers and patients. Am J Med 116: 559-560.

19. Chan MH, Chan PK, Griffith JF, Chan IH, Lit LC, et al. (2006) Steroid-induced osteonecrosis in severe acute respiratory syndrome: a retrospective analysis of biochemical markers of bone metabolism and corticosteroid therapy. Pathology 38: 229-235.

20. Chan MH, Wong VW, Wong CK, Chan PK, Chu CM, et al. (2004) Serum LD1 isoenzyme and blood lymphocyte subsets as prognostic indicators for severe acute respiratory syndrome. J Intern Med 255: 512-518.

21. Chan MS, Chan IY, Fung KH, Poon E, Yam LY, et al. (2004) High-resolution CT findings in patients with severe acute respiratory syndrome: a pattern-based approach. AJR Am J Roentgenol 182: 49-56.

22. Chan PK, Ip M, Ng KC, Rickjason CW, Wu A, et al. (2003) Severe acute respiratory syndrome-associated coronavirus infection. Emerg Infect Dis 9: 1453-1454.

23. Chan PK, Lim PL, Liu EY, Cheung JL, Leung DT, et al. (2005) Antibody avidity maturation during severe acute respiratory syndrome-associated coronavirus infection. J Infect Dis 192: 166-169.

24. Chan PK, Liu EY, Leung DT, Cheung JL, Ma CH, et al. (2005) Evaluation of a recombinant nucleocapsid protein-based assay for anti-SARS-CoV IgG detection. J Med Virol 75: 181-184.

25. Chan PK, Ng KC, Chan RC, Lam RK, Chow VC, et al. (2004) Immunofluorescence assay for serologic diagnosis of SARS. Emerg Infect Dis 10: 530-532.

26. Chan PK, To WK, Liu EY, Ng TK, Tam JS, et al. (2004) Evaluation of a peptide-based enzyme immunoassay for anti-SARS coronavirus IgG antibody. J Med Virol 74: 517-520.

27. Chan PK, To WK, Ng KC, Lam RK, Ng TK, et al. (2004) Laboratory diagnosis of SARS. Emerg Infect Dis 10: 825-831.

28. Chan SS, Leung DY, Wong EM, Tiwari AF, Wong DC, et al. (2006) Balancing infection control practices and family-centred care in a cohort of paediatric suspected severe acute respiratory syndrome patients in Hong Kong. J Paediatr Child Health 42: 20-27.

29. Chan SS, Leung GM, Tiwari AF, Salili F, Leung SS, et al. (2005) The impact of work-related risk on nurses during the SARS outbreak in Hong Kong. Fam Community Health 28: 274-287.

30. Chan SS, Mak PS, Shing KK, Chan PN, Ng WH, et al. (2005) Chest radiograph screening for severe acute respiratory syndrome in the ED. Am J Emerg Med 23: 525-530.

31. Chan SS, So WK, Wong DC, Lee AC, Tiwari A (2007) Improving older adults' knowledge and practice of preventive measures through a telephone health education during the SARS epidemic in Hong Kong: a pilot study. Int J Nurs Stud 44: 1120-1127.

32. Chan TY, Miu KY, Tsui CK, Yee KS, Chan MH (2004) A comparative study of clinical features and outcomes in young and older adults with severe acute respiratory syndrome. J Am Geriatr Soc 52: 1321-1325.

33. Chan VS, Chan KY, Chen Y, Poon LL, Cheung AN, et al. (2006) Homozygous L-SIGN (CLEC4M) plays a protective role in SARS coronavirus infection. Nat Genet 38: 38-46.

34. Chau PH, Yip PS (2003) Monitoring the severe acute respiratory syndrome epidemic and assessing effectiveness of interventions in Hong Kong Special Administrative Region. J Epidemiol Community Health 57: 766-769.

35. Chau TN, Lee PO, Choi KW, Lee CM, Ma KF, et al. (2004) Value of initial chest radiographs for predicting clinical outcomes in patients with severe acute respiratory syndrome. Am J Med 117: 249-254.

36. Che XY, Hao W, Wang YD, Di B, Yin K, et al. (2004) Nucleocapsid protein as early diagnostic marker for SARS. Emerg Infect Dis 10: 1947-1949.

37. Che XY, Qiu LW, Liao ZY, Wang YD, Wen K, et al. (2005) Antigenic cross-reactivity between severe acute respiratory syndrome-associated coronavirus and human coronaviruses 229E and OC43. J Infect Dis 191: 2033-2037.

38. Che XY, Qiu LW, Pan YX, Wen K, Hao W, et al. (2004) Sensitive and specific monoclonal antibody-based capture enzyme immunoassay for detection of nucleocapsid antigen in sera from patients with severe acute respiratory syndrome. J Clin Microbiol 42: 2629-2635.

39. Cheng FW, Ng PC, Chiu WK, Chu WC, Li AM, et al. (2005) A case-control study of SARS versus community acquired pneumonia. Arch Dis Child 90: 747-749.

40. Cheng PK, Wong DA, Tong LK, Ip SM, Lo AC, et al. (2004) Viral shedding patterns of coronavirus in patients with probable severe acute respiratory syndrome. Lancet 363: 1699-1700.

41. Cheng SK, Sheng B, Lau KK, Wong CW, Ng YK, et al. (2004) Adjustment outcomes in Chinese patients following one-month recovery from severe acute respiratory syndrome in Hong Kong. J Nerv Ment Dis 192: 868-871.

42. Cheng SK, Tsang JS, Ku KH, Wong CW, Ng YK (2004) Psychiatric complications in patients with severe acute respiratory syndrome (SARS) during the acute treatment phase: a series of 10 cases. Br J Psychiatry 184: 359-360.

43. Cheng SK, Wong CW, Tsang J, Wong KC (2004) Psychological distress and negative appraisals in survivors of severe acute respiratory syndrome (SARS). Psychol Med 34: 1187-1195.

44. Cheng SKW, Chong GHC, Chang SSY, Wong CW, Wong CSY, et al. (2006) Adjustment to severe acute respiratory syndrome (SARS): roles of appraisal and post-traumatic growth. Psychol Health 21: 301-317.

45. Cheng VC, Hung IF, Tang BS, Chu CM, Wong MM, et al. (2004) Viral replication in the nasopharynx is associated with diarrhea in patients with severe acute respiratory syndrome. Clin Infect Dis 38: 467-475.

46. Cheng WT, Li CK, Leung TF, Li AM, Hon KL, et al. (2004) Ribavirin for SARS in children. Clin Pediatr (Phila) 43: 193-196.

47. Cheng Y, Wong R, Soo YO, Wong WS, Lee CK, et al. (2005) Use of convalescent plasma therapy in SARS patients in Hong Kong. Eur J Clin Microbiol Infect Dis 24: 44-46.

48. Cheung OY, Chan JW, Ng CK, Koo CK (2004) The spectrum of pathological changes in severe acute respiratory syndrome (SARS). Histopathology 45: 119-124.

49. Cheung TM, Yam LY, So LK, Lau AC, Poon E, et al. (2004) Effectiveness of noninvasive positive pressure ventilation in the treatment of acute respiratory failure in severe acute respiratory syndrome. Chest 126: 845-850.

50. Chiu WK, Cheung PC, Ng KL, Ip PL, Sugunan VK, et al. (2003) Severe acute respiratory syndrome in children: experience in a regional hospital in Hong Kong. Pediatr Crit Care Med 4: 279-283.

51. Choi KW, Chau TN, Tsang O, Tso E, Chiu MC, et al. (2003) Outcomes and prognostic factors in 267 patients with severe acute respiratory syndrome in Hong Kong. Ann Intern Med 139: 715-723.

52. Chong WP, Ip WK, Tso GH, Ng MW, Wong WH, et al. (2006) The interferon gamma gene polymorphism +874 A/T is associated with severe acute respiratory syndrome. BMC Infect Dis 6: 82.

53. Chow SC, Ho CY, Tam TT, Wu C, Cheung T, et al. (2006) Specific epitopes of the structural and hypothetical proteins elicit variable humoral responses in SARS patients. J Clin Pathol 59: 468-476.

54. Chu CM, Cheng VC, Hung IF, Chan KS, Tang BS, et al. (2005) Viral load distribution in SARS outbreak. Emerg Infect Dis 11: 1882-1886.

55. Chu CM, Cheng VC, Hung IF, Wong MM, Chan KH, et al. (2004) Role of lopinavir/ritonavir in the treatment of SARS: initial virological and clinical findings. Thorax 59: 252-256.

56. Chu CM, Leung WS, Cheng VC, Chan KH, Lin AW, et al. (2005) Duration of RT-PCR positivity in severe acute respiratory syndrome. Eur Respir J 25: 12-14.

57. Chu CM, Leung YY, Hui JY, Hung IF, Chan VL, et al. (2004) Spontaneous pneumomediastinum in patients with severe acute respiratory syndrome. Eur Respir J 23: 802-804.

58. Chu CM, Poon LL, Cheng VC, Chan KS, Hung IF, et al. (2004) Initial viral load and the outcomes of SARS. CMAJ 171: 1349-1352.

59. Chu KH, Tsang WK, Tang CS, Lam MF, Lai FM, et al. (2005) Acute renal impairment in coronavirus-associated severe acute respiratory syndrome. Kidney Int 67: 698-705.

60. Chu WC, Li AM, Ng AW, So HK, Lam WW, et al. (2006) Thin-Section CT 12 Months After the Diagnosis of Severe Acute Respiratory Syndrome in Pediatric Patients. AJR Am J Roentgenol 186: 1707-1714.

61. Chua SE, Cheung V, Cheung C, McAlonan GM, Wong JW, et al. (2004) Psychological effects of the SARS outbreak in Hong Kong on high-risk health care workers. Can J Psychiatry 49: 391-393.

62. Chua SE, Cheung V, McAlonan GM, Cheung C, Wong JW, et al. (2004) Stress and psychological impact on SARS patients during the outbreak. Can J Psychiatry 49: 385-390.

63. Chung GT, Chiu RW, Cheung JL, Jin Y, Chim SS, et al. (2005) A simple and rapid approach for screening of SARS-coronavirus genotypes: an evaluation study. BMC Infect Dis 5: 87.

64. Chung JW, Wong TK, Chang KK, Chow CB, Chung BP, et al. (2004) Rapid assessment of a helpdesk service supporting severe acute respiratory syndrome patients and their relatives. J Clin Nurs 13: 748-755.

65. Derrick JL, Gomersall CD (2004) Surgical helmets and SARS infection. Emerg Infect Dis 10: 277-279.

66. Derrick JL, Gomersall CD (2005) Protecting healthcare staff from severe acute respiratory syndrome: filtration capacity of multiple surgical masks. J Hosp Infect 59: 365-368.

67. Di B, Hao W, Gao Y, Wang M, Wang YD, et al. (2005) Monoclonal antibody-based antigen capture enzyme-linked immunosorbent assay reveals high sensitivity of the nucleocapsid protein in acute-phase sera of severe acute respiratory syndrome patients. Clin Diagn Lab Immunol 12: 135-140.

68. Donnelly CA, Ghani AC, Leung GM, Hedley AJ, Fraser C, et al. (2003) Epidemiological determinants of spread of causal agent of severe acute respiratory syndrome in Hong Kong. Lancet 361: 1761-1766.

69. Drosten C, Chiu LL, Panning M, Leong HN, Preiser W, et al. (2004) Evaluation of advanced reverse transcription-PCR assays and an alternative PCR target region for detection of severe acute respiratory syndrome-associated coronavirus. J Clin Microbiol 42: 2043-2047.

70. Drosten C, Doerr HW, Lim W, Stohr K, Niedrig M (2004) SARS molecular detection external quality assurance. Emerg Infect Dis 10: 2200-2203.

71. Farewell VT, Herzberg AM, James KW, Ho LM, Leung GM (2005) SARS incubation and quarantine times: when is an exposed individual known to be disease free? Stat Med 24: 3431-3445.

72. Gomersall CD, Joynt GM, Ho OM, Ip M, Yap F, et al. (2006) Transmission of SARS to healthcare workers. The experience of a Hong Kong ICU. Intensive Care Med 32: 564-569.

73. Gomersall CD, Joynt GM, Lam P, Li T, Yap F, et al. (2004) Short-term outcome of critically ill patients with severe acute respiratory syndrome. Intensive Care Med 30: 381-387.

74. Griffith JF, Antonio GE, Kumta SM, Hui DS, Wong JK, et al. (2005) Osteonecrosis of hip and knee in patients with severe acute respiratory syndrome treated with steroids. Radiology 235: 168-175.

75. Guan M, Chan KH, Peiris JSM, Kwan SW, Lam SY, et al. (2004) Evaluation and validation of an enzyme-linked immunosorbent assay and an immunochromatographic test for serological diagnosis of severe acute respiratory syndrome. Clin Diagn Lab Immunol 11: 699-703.

76. Guan Y, Peiris JS, Zheng B, Poon LL, Chan KH, et al. (2004) Molecular epidemiology of the novel coronavirus that causes severe acute respiratory syndrome. Lancet 363: 99-104.

77. Heung LC, Li T, Mak SK, Chan WM (2006) Prevalence of subclinical infection and transmission of severe acute respiratory syndrome (SARS) in a residential care home for the elderly. Hong Kong Med J 12: 201-207.

78. Ho AS, Sung JJ, Chan-Yeung M (2003) An outbreak of severe acute respiratory syndrome among hospital workers in a community hospital in Hong Kong. Ann Intern Med 139: 564-567.

79. Ho JC, Ooi GC, Mok TY, Chan JW, Hung I, et al. (2003) High-dose pulse versus nonpulse corticosteroid regimens in severe acute respiratory syndrome. Am J Respir Crit Care Med 168: 1449-1456.

80. Ho JC, Wu AY, Lam B, Ooi GC, Khong PL, et al. (2004) Pentaglobin in steroid-resistant severe acute respiratory syndrome. Int J Tuberc Lung Dis 8: 1173-1179.

81. Ho PL, Chau PH, Yip PS, Ooi GC, Khong PL, et al. (2005) A prediction rule for clinical diagnosis of severe acute respiratory syndrome. Eur Respir J 26: 474-479.

82. Ho SM, Kwong-Lo RS, Mak CW, Wong JS (2005) Fear of severe acute respiratory syndrome (SARS) among health care workers. J Consult Clin Psychol 73: 344-349.

83. Hon KL, Leung CW, Cheng WT, Chan PK, Chu WC, et al. (2003) Clinical presentations and outcome of severe acute respiratory syndrome in children. Lancet 361: 1701-1703.

84. Hui DS, Joynt GM, Wong KT, Gomersall CD, Li TS, et al. (2005) Impact of severe acute respiratory syndrome (SARS) on pulmonary function, functional capacity and quality of life in a cohort of survivors. Thorax 60: 401-409.

85. Hui DS, Wong KT, Antonio GE, Lee N, Wu A, et al. (2004) Severe acute respiratory syndrome: correlation between clinical outcome and radiologic features. Radiology 233: 579-585.

86. Hui DS, Wong KT, Ko FW, Tam LS, Chan DP, et al. (2005) The 1-year impact of severe acute respiratory syndrome on pulmonary function, exercise capacity, and quality of life in a cohort of survivors. Chest 128: 2247-2261.

87. Hui JY, Hon TY, Yang MK, Cho DH, Luk WH, et al. (2004) High-resolution computed tomography is useful for early diagnosis of severe acute respiratory syndrome-associated coronavirus pneumonia in patients with normal chest radiographs. J Comput Assist Tomogr 28: 1-9.

88. Hui RK, Zeng F, Chan CM, Yuen KY, Peiris JS, et al. (2004) Reverse transcriptase PCR diagnostic assay for the coronavirus associated with severe acute respiratory syndrome. J Clin Microbiol 42: 1994-1999.

89. Hung IF, Cheng VC, Wu AK, Tang BS, Chan KH, et al. (2004) Viral loads in clinical specimens and SARS manifestations. Emerg Infect Dis 10: 1550-1557.

90. Ip M, Chan PK, Lee N, Wu A, Ng TK, et al. (2004) Seroprevalence of antibody to severe acute respiratory syndrome (SARS)-associated coronavirus among health care workers in SARS and non-SARS medical wards. Clin Infect Dis 38: e116-118.

91. Ip WK, Chan KH, Law HK, Tso GH, Kong EK, et al. (2005) Mannose-binding lectin in severe acute respiratory syndrome coronavirus infection. J Infect Dis 191: 1697-1704.

92. Jones BM, Ma ES, Peiris JS, Wong PC, Ho JC, et al. (2004) Prolonged disturbances of in vitro cytokine production in patients with severe acute respiratory syndrome (SARS) treated with ribavirin and steroids. Clin Exp Immunol 135: 467-473.

93. Joynt GM, Antonio GE, Lam P, Wong KT, Li T, et al. (2004) Late-stage adult respiratory distress syndrome caused by severe acute respiratory syndrome: abnormal findings at thin-section CT. Radiology 230: 339-346.

94. Karlberg J, Chong DS, Lai WY (2004) Do men have a higher case fatality rate of severe acute respiratory syndrome than women do? Am J Epidemiol 159: 229-231.

95. Kuiken T, Fouchier RAM, Schutten M, Rimmelzwaan GF, van Amerongen G, et al. (2003) Newly discovered coronavirus as the primary cause of severe acute respiratory syndrome. Lancet 362: 263-270.

96. Kwan AC, Chau TN, Tong WL, Tsang OT, Tso EY, et al. (2005) Severe acute respiratory syndrome-related diarrhea. J Gastroenterol Hepatol 20: 606-610.

97. Kwok KO, Leung GM, Lam WY, Riley S (2007) Using models to identify routes of nosocomial infection: a large hospital outbreak of SARS in Hong Kong. Proc Biol Sci 274: 611-617.

98. Lai PC, Wong CM, Hedley AJ, Lo SV, Leung PY, et al. (2004) Understanding the spatial clustering of severe acute respiratory syndrome (SARS) in Hong Kong. Environ Health Perspect 112: 1550-1556.

99. Lai TS, Keung Ng T, Seto WH, Yam L, Law KI, et al. (2005) Low prevalence of subclinical severe acute respiratory syndrome-associated coronavirus infection among hospital healthcare workers in Hong Kong. Scand J Infect Dis 37: 500-503.

100. Lam CM, Wong SF, Leung TN, Chow KM, Yu WC, et al. (2004) A case-controlled study comparing clinical course and outcomes of pregnant and non-pregnant women with severe acute respiratory syndrome. BJOG 111: 771-774.

101. Lau AC, So LK, Miu FP, Yung RW, Poon E, et al. (2004) Outcome of coronavirus-associated severe acute respiratory syndrome using a standard treatment protocol. Respirology 9: 173-183.

102. Lau EM, Chan FW, Hui DS, Wu AK, Leung PC (2005) Reduced bone mineral density in male Severe Acute Respiratory Syndrome (SARS) patients in Hong Kong. Bone 37: 420-424.

103. Lau HM, Lee EW, Wong CN, Ng GY, Jones AY, et al. (2005) The impact of severe acute respiratory syndrome on the physical profile and quality of life. Arch Phys Med Rehabil 86: 1134-1140.

104. Lau HM, Ng GY, Jones AY, Lee EW, Siu EH, et al. (2005) A randomised controlled trial of the effectiveness of an exercise training program in patients recovering from severe acute respiratory syndrome. Aust J Physiother 51: 213-219.

105. Lau JT, Fung KS, Wong TW, Kim JH, Wong E, et al. (2004) SARS transmission among hospital workers in Hong Kong. Emerg Infect Dis 10: 280-286.

106. Lau JT, Lau M, Kim JH, Tsui HY, Tsang T, et al. (2004) Probable secondary infections in households of SARS patients in Hong Kong. Emerg Infect Dis 10: 235-243.

107. Lau JT, Leung PC, Wong EL, Fong C, Cheng KF, et al. (2005) The use of an herbal formula by hospital care workers during the severe acute respiratory syndrome epidemic in Hong Kong to prevent severe acute respiratory syndrome transmission, relieve influenza-related symptoms, and improve quality of life: a prospective cohort study. J Altern Complement Med 11: 49-55.

108. Lau JT, Tsui H, Lau M, Yang X (2004) SARS transmission, risk factors, and prevention in Hong Kong. Emerg Infect Dis 10: 587-592.

109. Lau JT, Yang X, Leung PC, Chan L, Wong E, et al. (2004) SARS in three categories of hospital workers, Hong Kong. Emerg Infect Dis 10: 1399-1404.

110. Lau JT, Yang X, Pang E, Tsui HY, Wong E, et al. (2005) SARS-related perceptions in Hong Kong. Emerg Infect Dis 11: 417-424.

111. Lau JT, Yang X, Tsui H, Kim JH (2003) Monitoring community responses to the SARS epidemic in Hong Kong: from day 10 to day 62. J Epidemiol Community Health 57: 864-870.

112. Lau JT, Yang X, Tsui H, Pang E, Kim JH (2004) SARS preventive and risk behaviours of Hong Kong air travellers. Epidemiol Infect 132: 727-736.

113. Lau JT, Yang X, Tsui HY, Kim JH (2005) Impacts of SARS on health-seeking behaviors in general population in Hong Kong. Prev Med 41: 454-462.

114. Lau JT, Yang X, Tsui HY, Pang E (2004) SARS related preventive and risk behaviours practised by Hong Kong-mainland China cross border travellers during the outbreak of the SARS epidemic in Hong Kong. J Epidemiol Community Health 58: 988-996.

115. Lau JT, Yang X, Tsui HY, Pang E, Wing YK (2006) Positive mental health-related impacts of the SARS epidemic on the general public in Hong Kong and their associations with other negative impacts. J Infect 53: 114-124.

116. Lau JT, Yang X, Wong E, Tsui H (2006) Prevalence and factors associated with social avoidance of recovered SARS patients in the Hong Kong general population. Health Educ Res 21: 662-673.

117. Lau LT, Fung YWW, Wong FPF, Lin SSW, Wang CR, et al. (2003) A real-time PCR for SARS-coronavirus incorporating target gene pre-amplification. Biochem Biophys Res Commun 312: 1290-1296.

118. Lau SK, Che XY, Woo PC, Wong BH, Cheng VC, et al. (2005) SARS coronavirus detection methods. Emerg Infect Dis 11: 1108-1111.

119. Lau SK, Woo PC, Wong BH, Tsoi HW, Woo GK, et al. (2004) Detection of severe acute respiratory syndrome (SARS) coronavirus nucleocapsid protein in sars patients by enzyme-linked immunosorbent assay. J Clin Microbiol 42: 2884-2889.

120. Lee AM, Wong JG, McAlonan GM, Cheung V, Cheung C, et al. (2007) Stress and psychological distress among SARS survivors 1 year after the outbreak. Can J Psychiatry 52: 233-240.

121. Lee DT, Sahota D, Leung TN, Yip AS, Lee FF, et al. (2006) Psychological responses of pregnant women to an infectious outbreak: a case-control study of the 2003 SARS outbreak in Hong Kong. J Psychosom Res 61: 707-713.

122. Lee DT, Wing YK, Leung HC, Sung JJ, Ng YK, et al. (2004) Factors associated with psychosis among patients with severe acute respiratory syndrome: a case-control study. Clin Infect Dis 39: 1247-1249.

123. Lee N, Allen Chan KC, Hui DS, Ng EK, Wu A, et al. (2004) Effects of early corticosteroid treatment on plasma SARS-associated Coronavirus RNA concentrations in adult patients. J Clin Virol 31: 304-309.

124. Lee N, Chan PK, Ip M, Wong E, Ho J, et al. (2006) Anti-SARS-CoV IgG response in relation to disease severity of severe acute respiratory syndrome. J Clin Virol 35: 179-184.

125. Lee N, Hui D, Wu A, Chan P, Cameron P, et al. (2003) A major outbreak of severe acute respiratory syndrome in Hong Kong. N Engl J Med 348: 1986-1994.

126. Lee N, Rainer TH, Ip M, Zee B, Ng MH, et al. (2006) Role of laboratory variables in differentiating SARS-coronavirus from other causes of community-acquired pneumonia within the first 72 h of hospitalization. Eur J Clin Microbiol Infect Dis 25: 765-772.

127. Lee PP, Wong WH, Leung GM, Chiu SS, Chan KH, et al. (2006) Risk-stratified seroprevalence of severe acute respiratory syndrome coronavirus among children in Hong Kong. Pediatrics 117: e1156-1162.

128. Lee S, Chan LY, Chau AM, Kwok KP, Kleinman A (2005) The experience of SARS-related stigma at Amoy Gardens. Soc Sci Med 61: 2038-2046.

129. Lee TM, Chi I, Chung LW, Chou KL (2006) Ageing and psychological response during the post-SARS period. Aging Ment Health 10: 303-311.

130. Leung CW, Kwan YW, Ko PW, Chiu SS, Loung PY, et al. (2004) Severe acute respiratory syndrome among children. Pediatrics 113: e535-543.

131. Leung DT, Tam FC, Ma CH, Chan PK, Cheung JL, et al. (2004) Antibody response of patients with severe acute respiratory syndrome (SARS) targets the viral nucleocapsid. J Infect Dis 190: 379-386.

132. Leung DT, van Maren WW, Chan FK, Chan WS, Lo AW, et al. (2006) Extremely low exposure of a community to severe acute respiratory syndrome coronavirus: false seropositivity due to use of bacterially derived antigens. J Virol 80: 8920-8928.

133. Leung GM, Chung PH, Tsang T, Lim W, Chan SK, et al. (2004) SARS-CoV antibody prevalence in all Hong Kong patient contacts. Emerg Infect Dis 10: 1653-1656.

134. Leung GM, Hedley AJ, Ho LM, Chau P, Wong IO, et al. (2004) The epidemiology of severe acute respiratory syndrome in the 2003 Hong Kong epidemic: an analysis of all 1755 patients. Ann Intern Med 141: 662-673.

135. Leung GM, Ho LM, Chan SK, Ho SY, Bacon-Shone J, et al. (2005) Longitudinal assessment of community psychobehavioral responses during and after the 2003 outbreak of severe acute respiratory syndrome in Hong Kong. Clin Infect Dis 40: 1713-1720.

136. Leung GM, Lam TH, Ho LM, Ho SY, Chan BH, et al. (2003) The impact of community psychological responses on outbreak control for severe acute respiratory syndrome in Hong Kong. J Epidemiol Community Health 57: 857-863.

137. Leung GM, Quah S, Ho LM, Ho SY, Hedley AJ, et al. (2004) A tale of two cities: community psychobehavioral surveillance and related impact on outbreak control in Hong Kong and Singapore during the severe acute respiratory syndrome epidemic. Infect Control Hosp Epidemiol 25: 1033-1041.

138. Leung GM, Rainer TH, Lau FL, Wong IO, Tong A, et al. (2004) A clinical prediction rule for diagnosing severe acute respiratory syndrome in the emergency department. Ann Intern Med 141: 333-342.

139. Leung TF, Ng PC, Cheng FW, Lyon DJ, So KW, et al. (2004) Infection control for SARS in a tertiary paediatric centre in Hong Kong. J Hosp Infect 56: 215-222.

140. Leung TW, Wong KS, Hui AC, To KF, Lai ST, et al. (2005) Myopathic changes associated with severe acute respiratory syndrome: a postmortem case series. Arch Neurol 62: 1113-1117.

141. Leung WK, To KF, Chan PK, Chan HL, Wu AK, et al. (2003) Enteric involvement of severe acute respiratory syndrome-associated coronavirus infection. Gastroenterology 125: 1011-1017.

142. Li AM, Chan CH, Chan DF (2004) Long-term sequelae of SARS in children. Paediatr Respir Rev 5: 296-299.

143. Li AM, So HK, Chu W, Ng PC, Hon KL, et al. (2004) Radiological and pulmonary function outcomes of children with SARS. Pediatr Pulmonol 38: 427-433.

144. Li SS, Cheng CW, Fu CL, Chan YH, Lee MP, et al. (2003) Left ventricular performance in patients with severe acute respiratory syndrome: a 30-day echocardiographic follow-up study. Circulation 108: 1798-1803.

145. Li TS, Gomersall CD, Joynt GM, Chan DP, Leung P, et al. (2006) Long-term outcome of acute respiratory distress syndrome caused by severe acute respiratory syndrome (SARS): an observational study. Crit Care Resusc 8: 302-308.

146. Li Y, Duan S, Yu IT, Wong TW (2005) Multi-zone modeling of probable SARS virus transmission by airflow between flats in Block E, Amoy Gardens. Indoor Air 15: 96-111.

147. Li Y, Huang X, Yu IT, Wong TW, Qian H (2005) Role of air distribution in SARS transmission during the largest nosocomial outbreak in Hong Kong. Indoor Air 15: 83-95.

148. Li Y, Wong T, Chung J, Guo YP, Hu JY, et al. (2006) In vivo protective performance of N95 respirator and surgical facemask. Am J Ind Med 49: 1056-1065.

149. Li Y, Yu IT, Xu P, Lee JH, Wong TW, et al. (2004) Predicting super spreading events during the 2003 severe acute respiratory syndrome epidemics in Hong Kong and Singapore. Am J Epidemiol 160: 719-728.

150. Lin K, Yee-Tak Fong D, Zhu B, Karlberg J (2006) Environmental factors on the SARS epidemic: air temperature, passage of time and multiplicative effect of hospital infection. Epidemiol Infect 134: 223-230.

151. Lin L, Xu YJ, He DP, Han Y, Tang GH, et al. (2003) A retrospective study on clinical features of and treatment methods for 77 severe cases of SARS. Am J Chin Med 31: 821-839.

152. Lloyd-Smith JO, Galvani AP, Getz WM (2003) Curtailing transmission of severe acute respiratory syndrome within a community and its hospital. Proc Biol Sci 270: 1979-1989.

153. McAlonan GM, Lee AM, Cheung V, Cheung C, Tsang KW, et al. (2007) Immediate and sustained psychological impact of an emerging infectious disease outbreak on health care workers. Can J Psychiatry 52: 241-247.

154. Muller NL, Ooi GC, Khong PL, Zhou LJ, Tsang KWT, et al. (2004) High-resolution CT findings of severe acute respiratory syndrome at presentation and after admission. AJR Am J Roentgenol 182: 39-44.

155. Ng CK, Chan JW, Kwan TL, To TS, Chan YH, et al. (2004) Six month radiological and physiological outcomes in severe acute respiratory syndrome (SARS) survivors. Thorax 59: 889-891.

156. Ng DK, Chan CH, Lee RS, Leung LC (2005) Non-contact infrared thermometry temperature measurement for screening fever in children. Ann Trop Paediatr 25: 267-275.

157. Ng EK, Hui DS, Chan KC, Hung EC, Chiu RW, et al. (2003) Quantitative analysis and prognostic implication of SARS coronavirus RNA in the plasma and serum of patients with severe acute respiratory syndrome. Clin Chem 49: 1976-1980.

158. Ng MH, Lau KM, Li L, Cheng SH, Chan WY, et al. (2004) Association of human-leukocyte-antigen class I (B*0703) and class II (DRB1*0301) genotypes with susceptibility and resistance to the development of severe acute respiratory syndrome. J Infect Dis 190: 515-518.

159. Ng MW, Zhou G, Chong WP, Lee LW, Law HK, et al. (2007) The association of RANTES polymorphism with severe acute respiratory syndrome in Hong Kong and Beijing Chinese. BMC Infect Dis 7: 50.

160. Ng PC, Lam CW, Li AM, Wong CK, Cheng FW, et al. (2004) Inflammatory cytokine profile in children with severe acute respiratory syndrome. Pediatrics 113: e7-14.

161. Ng PC, Lam CW, Li AM, Wong CK, Leung TF, et al. (2005) Chemokine response in children with SARS. Arch Dis Child 90: 422-423.

162. Ng PC, So KW, Leung TF, Cheng FW, Lyon DJ, et al. (2003) Infection control for SARS in a tertiary neonatal centre. Arch Dis Child Fetal Neonatal Ed 88: F405-409.

163. Ng SM, Chan TH, Chan CL, Lee AM, Yau JK, et al. (2006) Group debriefing for people with chronic diseases during the SARS pandemic: Strength-Focused and Meaning-Oriented Approach for Resilience and Transformation (SMART). Community Ment Health J 42: 53-63.

164. Ng TW, Turinici G, Danchin A (2003) A double epidemic model for the SARS propagation. BMC Infect Dis 3: 19.

165. Ng WF, Wong SF, Lam A, Mak YF, Yao H, et al. (2006) The placentas of patients with severe acute respiratory syndrome: a pathophysiological evaluation. Pathology 38: 210-218.

166. Nicholls JM, Poon LL, Lee KC, Ng WF, Lai ST, et al. (2003) Lung pathology of fatal severe acute respiratory syndrome. Lancet 361: 1773-1778.

167. Olsen SJ, Chang HL, Cheung TY, Tang AF, Fisk TL, et al. (2003) Transmission of the severe acute respiratory syndrome on aircraft. N Engl J Med 349: 2416-2422.

168. Ooi CG, Khong PL, Ho JC, Lam B, Wong WM, et al. (2003) Severe acute respiratory syndrome: radiographic evaluation and clinical outcome measures. Radiology 229: 500-506.

169. Ooi CG, Khong PL, Lam B, Ho JC, Yiu WC, et al. (2003) Severe acute respiratory syndrome: relationship between radiologic and clinical parameters. Radiology 229: 492-499.

170. Ooi GC, Khong PL, Muller NL, Yiu WC, Zhou LJ, et al. (2004) Severe acute respiratory syndrome: temporal lung changes at thin-section CT in 30 patients. Radiology 230: 836-844.

171. Pang RT, Poon TC, Chan KC, Lee NL, Chiu RW, et al. (2006) Serum proteomic fingerprints of adult patients with severe acute respiratory syndrome. Clin Chem 52: 421-429.

172. Peiris JS, Chu CM, Cheng VC, Chan KS, Hung IF, et al. (2003) Clinical progression and viral load in a community outbreak of coronavirus-associated SARS pneumonia: a prospective study. Lancet 361: 1767-1772.

173. Peiris JS, Lai ST, Poon LL, Guan Y, Yam LY, et al. (2003) Coronavirus as a possible cause of severe acute respiratory syndrome. Lancet 361: 1319-1325.

174. Poon E, Liu KS, Cheong DL, Lee CK, Yam LY, et al. (2004) Impact of severe respiratory syndrome on anxiety levels of front-line health care workers. Hong Kong Med J 10: 325-330.

175. Poon LL, Chan KH, Wong OK, Cheung TK, Ng I, et al. (2004) Detection of SARS coronavirus in patients with severe acute respiratory syndrome by conventional and real-time quantitative reverse transcription-PCR assays. Clin Chem 50: 67-72.

176. Poon LL, Chan KH, Wong OK, Yam WC, Yuen KY, et al. (2003) Early diagnosis of SARS coronavirus infection by real time RT-PCR. J Clin Virol 28: 233-238.

177. Poon LL, Leung CS, Tashiro M, Chan KH, Wong BW, et al. (2004) Rapid detection of the severe acute respiratory syndrome (SARS) coronavirus by a loop-mediated isothermal amplification assay. Clin Chem 50: 1050-1052.

178. Poon LL, Wong BW, Chan KH, Leung CS, Yuen KY, et al. (2004) A one step quantitative RT-PCR for detection of SARS coronavirus with an internal control for PCR inhibitors. J Clin Virol 30: 214-217.

179. Poon LL, Wong BW, Chan KH, Ng SS, Yuen KY, et al. (2005) Evaluation of real-time reverse transcriptase PCR and real-time loop-mediated amplification assays for severe acute respiratory syndrome coronavirus detection. J Clin Microbiol 43: 3457-3459.

180. Poon LL, Wong OK, Chan KH, Luk W, Yuen KY, et al. (2003) Rapid diagnosis of a coronavirus associated with severe acute respiratory syndrome (SARS). Clin Chem 49: 953-955.

181. Poon TC, Chan KC, Ng PC, Chiu RW, Ang IL, et al. (2004) Serial analysis of plasma proteomic signatures in pediatric patients with severe acute respiratory syndrome and correlation with viral load. Clin Chem 50: 1452-1455.

182. Rainer TH, Cameron PA, Smit D, Ong KL, Hung AN, et al. (2003) Evaluation of WHO criteria for identifying patients with severe acute respiratory syndrome out of hospital: prospective observational study. BMJ 326: 1354-1358.

183. Rainer TH, Chan PK, Ip M, Lee N, Hui DS, et al. (2004) The spectrum of severe acute respiratory syndrome-associated coronavirus infection. Ann Intern Med 140: 614-619.

184. Rainer TH, Lee N, Ip M, Galvani AP, Antonio GE, et al. (2007) Features discriminating SARS from other severe viral respiratory tract infections. Eur J Clin Microbiol Infect Dis 26: 121-129.

185. Ren Y, He QY, Fan J, Jones B, Zhou Y, et al. (2004) The use of proteomics in the discovery of serum biomarkers from patients with severe acute respiratory syndrome. Proteomics 4: 3477-3484.

186. Riley S, Fraser C, Donnelly CA, Ghani AC, Abu-Raddad LJ, et al. (2003) Transmission dynamics of the etiological agent of SARS in Hong Kong: impact of public health interventions. Science 300: 1961-1966.

187. Seto WH, Tsang D, Yung RW, Ching TY, Ng TK, et al. (2003) Effectiveness of precautions against droplets and contact in prevention of nosocomial transmission of severe acute respiratory syndrome (SARS). Lancet 361: 1519-1520.

188. Sheng B, Cheng SK, Lau KK, Li HL, Chan EL (2005) The effects of disease severity, use of corticosteroids and social factors on neuropsychiatric complaints in severe acute respiratory syndrome (SARS) patients at acute and convalescent phases. Eur Psychiatry 20: 236-242.

189. Sihoe AD, Wong RH, Lee AT, Lau LS, Leung NY, et al. (2004) Severe acute respiratory syndrome complicated by spontaneous pneumothorax. Chest 125: 2345-2351.

190. Small M, Shi PL, Tse CK (2004) Plausible models for propagation of the SARS virus. Ieice Transactions On Fundamentals Of Electronics Communications And Computer Sciences E87A: 2379-2386.

191. Small M, Tse CK (2005) Clustering model for transmission of the SARS virus: application to epidemic control and risk assessment. Physica A-Statistical Mechanics And Its Applications 351: 499-511.

192. Small M, Tse CK (2005) Small world and scale free model of transmission of SARS. International Journal Of Bifurcation And Chaos 15: 1745-1755.

193. Small M, Tse CK, Walker DM (2006) Super-spreaders and the rate of transmission of the SARS virus. Physica D-Nonlinear Phenomena 215: 146-158.

194. So LK, Lau AC, Yam LY, Cheung TM, Poon E, et al. (2003) Development of a standard treatment protocol for severe acute respiratory syndrome. Lancet 361: 1615-1617.

195. So WK, Chan SS, Lee AC, Tiwari AF (2004) The knowledge level and precautionary measures taken by older adults during the SARS outbreak in Hong Kong. Int J Nurs Stud 41: 901-909.

196. Soo YO, Cheng Y, Wong R, Hui DS, Lee CK, et al. (2004) Retrospective comparison of convalescent plasma with continuing high-dose methylprednisolone treatment in SARS patients. Clin Microbiol Infect 10: 676-678.

197. Stockman LJ, Massoudi MS, Helfand R, Erdman D, Siwek AM, et al. (2007) Severe acute respiratory syndrome in children. Pediatr Infect Dis J 26: 68-74.

198. Sung JJ, Wu A, Joynt GM, Yuen KY, Lee N, et al. (2004) Severe acute respiratory syndrome: report of treatment and outcome after a major outbreak. Thorax 59: 414-420.

199. Tam CW, Pang EP, Lam LC, Chiu HF (2004) Severe acute respiratory syndrome (SARS) in Hong Kong in 2003: stress and psychological impact among frontline healthcare workers. Psychol Med 34: 1197-1204.

200. Tam KP, Lau IYM, Chiu CY (2004) Biases in the perceived prevalence and motives of severe acute respiratory syndrome prevention behaviors among Chinese high school students in Hong Kong. Asian Journal Of Social Psychology 7: 67-81.

201. Tang CS, Wong CY (2003) An outbreak of the severe acute respiratory syndrome: predictors of health behaviors and effect of community prevention measures in Hong Kong, China. Am J Public Health 93: 1887-1888.

202. Tang CS, Wong CY (2004) Factors influencing the wearing of facemasks to prevent the severe acute respiratory syndrome among adult Chinese in Hong Kong. Prev Med 39: 1187-1193.

203. Tang CS, Wong CY (2005) Psychosocial factors influencing the practice of preventive behaviors against the severe acute respiratory syndrome among older Chinese in Hong Kong. J Aging Health 17: 490-506.

204. Tang JW, Cheung JL, Chu IM, Ip M, Hui M, et al. (2007) Characterizing 56 complete SARS-CoV S-gene sequences from Hong Kong. J Clin Virol 38: 19-26.

205. Tang JW, Cheung JL, Chu IM, Sung JJ, Peiris M, et al. (2006) The large 386-nt deletion in SARS-associated coronavirus: evidence for quasispecies? J Infect Dis 194: 808-813.

206. Tang NL, Chan PK, Wong CK, To KF, Wu AK, et al. (2005) Early enhanced expression of interferon-inducible protein-10 (CXCL-10) and other chemokines predicts adverse outcome in severe acute respiratory syndrome. Clin Chem 51: 2333-2340.

207. Temperton NJ, Chan PK, Simmons G, Zambon MC, Tedder RS, et al. (2005) Longitudinally profiling neutralizing antibody response to SARS coronavirus with pseudotypes. Emerg Infect Dis 11: 411-416.

208. Tiwari A, Chan S, Wong A, Tai J, Cheng K, et al. (2003) Severe acute respiratory syndrome (SARS) in Hong Kong: patients' experiences. Nurs Outlook 51: 212-219.

209. Tsang KW, Ho PL, Ooi GC, Yee WK, Wang T, et al. (2003) A cluster of cases of severe acute respiratory syndrome in Hong Kong. N Engl J Med 348: 1977-1985.

210. Tsang OT, Chau TN, Choi KW, Tso EY, Lim W, et al. (2003) Coronavirus-positive nasopharyngeal aspirate as predictor for severe acute respiratory syndrome mortality. Emerg Infect Dis 9: 1381-1387.

211. Tse GM, Hui PK, Ma TK, Lo AW, To KF, et al. (2004) Sputum cytology of patients with severe acute respiratory syndrome (SARS). J Clin Pathol 57: 256-259.

212. Tse GM, To KF, Chan PK, Lo AW, Ng KC, et al. (2004) Pulmonary pathological features in coronavirus associated severe acute respiratory syndrome (SARS). J Clin Pathol 57: 260-265.

213. Tsui PT, Kwok ML, Yuen H, Lai ST (2003) Severe acute respiratory syndrome: clinical outcome and prognostic correlates. Emerg Infect Dis 9: 1064-1069.

214. Wong CK, Lam CW, Wu AK, Ip WK, Lee NL, et al. (2004) Plasma inflammatory cytokines and chemokines in severe acute respiratory syndrome. Clin Exp Immunol 136: 95-103.

215. Wong CY, Tang CS (2005) Practice of habitual and volitional health behaviors to prevent severe acute respiratory syndrome among Chinese adolescents in Hong Kong. J Adolesc Health 36: 193-200.

216. Wong JG, Cheung EP, Cheung V, Cheung C, Chan MT, et al. (2004) Psychological responses to the SARS outbreak in healthcare students in Hong Kong. Med Teach 26: 657-659.

217. Wong KT, Antonio GE, Hui DS, Lee N, Yuen EH, et al. (2003) Severe acute respiratory syndrome: radiographic appearances and pattern of progression in 138 patients. Radiology 228: 401-406.

218. Wong KT, Antonio GE, Hui DS, Lee N, Yuen EH, et al. (2003) Thin-section CT of severe acute respiratory syndrome: evaluation of 73 patients exposed to or with the disease. Radiology 228: 395-400.

219. Wong RS, Wu A, To KF, Lee N, Lam CW, et al. (2003) Haematological manifestations in patients with severe acute respiratory syndrome: retrospective analysis. BMJ 326: 1358-1362.

220. Wong SC, Chan JK, Lee KC, Lo ES, Tsang DN (2005) Development of a quantitative assay for SARS coronavirus and correlation of GAPDH mRNA with SARS coronavirus in clinical specimens. J Clin Pathol 58: 276-280.

221. Wong SF, Chow KM, Leung TN, Ng WF, Ng TK, et al. (2004) Pregnancy and perinatal outcomes of women with severe acute respiratory syndrome. Am J Obstet Gynecol 191: 292-297.

222. Wong TK, Chung JW, Li Y, Chan WF, Ching PT, et al. (2004) Effective personal protective clothing for health care workers attending patients with severe acute respiratory syndrome. Am J Infect Control 32: 90-96.

223. Wong TW, Gao Y, Tam WWS (2007) Anxiety among university students during the SARS epidemic in Hong Kong. Stress And Health 23: 31-35.

224. Wong TW, Lee CK, Tam W, Lau JT, Yu TS, et al. (2004) Cluster of SARS among medical students exposed to single patient, Hong Kong. Emerg Infect Dis 10: 269-276.

225. Wong TW, Tam WW (2005) Handwashing practice and the use of personal protective equipment among medical students after the SARS epidemic in Hong Kong. Am J Infect Control 33: 580-586.

226. Wong TW, Yau JK, Chan CL, Kwong RS, Ho SM, et al. (2005) The psychological impact of severe acute respiratory syndrome outbreak on healthcare workers in emergency departments and how they cope. Eur J Emerg Med 12: 13-18.

227. Wong WC, Lee A, Tsang KK, Wong SY (2004) How did general practitioners protect themselves, their family, and staff during the SARS epidemic in Hong Kong? J Epidemiol Community Health 58: 180-185.

228. Wong WN, Sek AC, Lau RF, Li KM, Leung JK, et al. (2003) Accuracy of clinical diagnosis versus the World Health Organization case definition in the Amoy Garden SARS cohort. CJEM 5: 384-391.

229. Wong WN, Sek AC, Lau RF, Li KM, Leung JK, et al. (2004) Early clinical predictors of severe acute respiratory syndrome in the emergency department. CJEM 6: 12-21.

230. Woo PC, Lau SK, Tsoi HW, Chan KH, Wong BH, et al. (2004) Relative rates of non-pneumonic SARS coronavirus infection and SARS coronavirus pneumonia. Lancet 363: 841-845.

231. Woo PC, Lau SK, Wong BH, Chan KH, Chu CM, et al. (2004) Longitudinal profile of immunoglobulin G (IgG), IgM, and IgA antibodies against the severe acute respiratory syndrome (SARS) coronavirus nucleocapsid protein in patients with pneumonia due to the SARS coronavirus. Clin Diagn Lab Immunol 11: 665-668.

232. Woo PC, Lau SK, Wong BH, Tsoi HW, Fung AM, et al. (2004) Detection of specific antibodies to severe acute respiratory syndrome (SARS) coronavirus nucleocapsid protein for serodiagnosis of SARS coronavirus pneumonia. J Clin Microbiol 42: 2306-2309.

233. Woo PC, Lau SK, Wong BH, Tsoi HW, Fung AM, et al. (2005) Differential sensitivities of severe acute respiratory syndrome (SARS) coronavirus spike polypeptide enzyme-linked immunosorbent assay (ELISA) and SARS coronavirus nucleocapsid protein ELISA for serodiagnosis of SARS coronavirus pneumonia. J Clin Microbiol 43: 3054-3058.

234. Wu KK, Chan SK, Ma TM (2005) Posttraumatic stress after SARS. Emerg Infect Dis 11: 1297-1300.

235. Wu KK, Chan SK, Ma TM (2005) Posttraumatic stress, anxiety, and depression in survivors of severe acute respiratory syndrome (SARS). J Trauma Stress 18: 39-42.

236. Yam LY, Chan AY, Cheung TM, Tsui EL, Chan JC, et al. (2005) Non-invasive versus invasive mechanical ventilation for respiratory failure in severe acute respiratory syndrome. Chin Med J (Engl) 118: 1413-1421.

237. Yam LY, Lau AC, Lai FY, Shung E, Chan J, et al. (2007) Corticosteroid treatment of severe acute respiratory syndrome in Hong Kong. J Infect 54: 28-39.

238. Yam WC, Chan KH, Chow KH, Poon LL, Lam HY, et al. (2005) Clinical evaluation of real-time PCR assays for rapid diagnosis of SARS coronavirus during outbreak and post-epidemic periods. J Clin Virol 33: 19-24.

239. Yam WC, Chan KH, Poon LL, Guan Y, Yuen KY, et al. (2003) Evaluation of reverse transcription-PCR assays for rapid diagnosis of severe acute respiratory syndrome associated with a novel coronavirus. J Clin Microbiol 41: 4521-4524.

240. Yip HK, Tsang PC, Samaranayake LP, Li AH (2007) Knowledge of and attitudes toward severe acute respiratory syndrome among a cohort of dental patients in Hong Kong following a major local outbreak. Community Dent Health 24: 43-48.

241. Yip SP, To SS, Leung PH, Cheung TS, Cheng PK, et al. (2005) Use of dual TaqMan probes to increase the sensitivity of 1-step quantitative reverse transcription-PCR: application to the detection of SARS coronavirus. Clin Chem 51: 1885-1888.

242. Yip TT, Chan JW, Cho WC, Wang Z, Kwan TL, et al. (2005) Protein chip array profiling analysis in patients with severe acute respiratory syndrome identified serum amyloid a protein as a biomarker potentially useful in monitoring the extent of pneumonia. Clin Chem 51: 47-55.

243. Yu CC, Li AM, So RC, McManus A, Ng PC, et al. (2006) Longer term follow up of aerobic capacity in children affected by severe acute respiratory syndrome (SARS). Thorax 61: 240-246.

244. Yu CM, Wong RS, Wu EB, Kong SL, Wong J, et al. (2006) Cardiovascular complications of severe acute respiratory syndrome. Postgrad Med J 82: 140-144.

245. Yu HYR, Ho SC, So KFE, Lo YL (2005) Short communication: The psychological burden experienced by Hong Kong midlife women during the SARS epidemic. Stress And Health 21: 177-184.

246. Yu IT, Li Y, Wong TW, Tam W, Chan AT, et al. (2004) Evidence of airborne transmission of the severe acute respiratory syndrome virus. N Engl J Med 350: 1731-1739.

247. Yu IT, Wong TW, Chiu YL, Lee N, Li Y (2005) Temporal-spatial analysis of severe acute respiratory syndrome among hospital inpatients. Clin Infect Dis 40: 1237-1243.

248. Yu IT, Xie ZH, Tsoi KK, Chiu YL, Lok SW, et al. (2007) Why did outbreaks of severe acute respiratory syndrome occur in some hospital wards but not in others? Clin Infect Dis 44: 1017-1025.

249. Yuan FF, Tanner J, Chan PK, Biffin S, Dyer WB, et al. (2005) Influence of FcgammaRIIA and MBL polymorphisms on severe acute respiratory syndrome. Tissue Antigens 66: 291-296.

250. Yuen KS, Chan WM, Fan DS, Chong KK, Sung JJ, et al. (2004) Ocular screening in severe acute respiratory syndrome. Am J Ophthalmol 137: 773-774.

251. Zheng BJ, Wong KH, Zhou J, Wong KL, Young BW, et al. (2004) SARS-related virus predating SARS outbreak, Hong Kong. Emerg Infect Dis 10: 176-178.

252. Zhong X, Yang H, Guo ZF, Sin WY, Chen W, et al. (2005) B-cell responses in patients who have recovered from severe acute respiratory syndrome target a dominant site in the S2 domain of the surface spike glycoprotein. J Virol 79: 3401-3408.

253. Zhou G, Yan G (2003) Severe acute respiratory syndrome epidemic in Asia. Emerg Infect Dis 9: 1608-1610.

254. Avendano M, Derkach P, Swan S (2003) Clinical course and management of SARS in health care workers in Toronto: a case series. CMAJ 168: 1649-1660.

255. Bitar R, Weiser WJ, Avendano M, Derkach P, Low DE, et al. (2004) Chest radiographic manifestations of severe acute respiratory syndrome in health care workers: the Toronto experience. AJR Am J Roentgenol 182: 45-48.

256. Bitnun A, Allen U, Heurter H, King SM, Opavsky MA, et al. (2003) Children hospitalized with severe acute respiratory syndrome-related illness in Toronto. Pediatrics 112: e261.

257. Blendon RJ, Benson JM, DesRoches CM, Raleigh E, Taylor-Clark K (2004) The public's response to severe acute respiratory syndrome in Toronto and the United States. Clin Infect Dis 38: 925-931.

258. Booth CM, Matukas LM, Tomlinson GA, Rachlis AR, Rose DB, et al. (2003) Clinical features and short-term outcomes of 144 patients with SARS in the greater Toronto area. JAMA 289: 2801-2809.

259. Booth TF, Kournikakis B, Bastien N, Ho J, Kobasa D, et al. (2005) Detection of airborne severe acute respiratory syndrome (SARS) coronavirus and environmental contamination in SARS outbreak units. J Infect Dis 191: 1472-1477.

260. Caputo KM, Byrick R, Chapman MG, Orser BJ, Orser BA (2006) Intubation of SARS patients: infection and perspectives of healthcare workers. Can J Anaesth 53: 122-129.

261. Choi BCK, Pak AWP (2003) A simple approximate mathematical model to predict the number of severe acute respiratory syndrome cases and deaths. J Epidemiol Community Health 57: 831-835.

262. Christian MD, Loutfy M, McDonald LC, Martinez KF, Ofner M, et al. (2004) Possible SARS coronavirus transmission during cardiopulmonary resuscitation. Emerg Infect Dis 10: 287-293.

263. Dimoulas P, Green KA, Shigayeva A, Aquino M, McGeer A, et al. (2005) Patient contact recall after SARS exposure. Emerg Infect Dis 11: 625-628.

264. Farcas GA, Poutanen SM, Mazzulli T, Willey BM, Butany J, et al. (2005) Fatal severe acute respiratory syndrome is associated with multiorgan involvement by coronavirus. J Infect Dis 191: 193-197.

265. Fowler RA, Guest CB, Lapinsky SE, Sibbald WJ, Louie M, et al. (2004) Transmission of severe acute respiratory syndrome during intubation and mechanical ventilation. Am J Respir Crit Care Med 169: 1198-1202.

266. Fowler RA, Lapinsky SE, Hallett D, Detsky AS, Sibbald WJ, et al. (2003) Critically ill patients with severe acute respiratory syndrome. JAMA 290: 367-373.

267. Grace SL, Hershenfield K, Robertson E, Stewart DE (2004) Factors affecting perceived risk of contracting severe acute respiratory syndrome among academic physicians. Infect Control Hosp Epidemiol 25: 1111-1113.

268. Grace SL, Hershenfield K, Robertson E, Stewart DE (2005) The occupational and psychosocial impact of SARS on academic physicians in three affected hospitals. Psychosomatics 46: 385-391.

269. Grinblat L, Shulman H, Glickman A, Matukas L, Paul N (2003) Severe acute respiratory syndrome: radiographic review of 40 probable cases in Toronto, Canada. Radiology 228: 802-809.

270. Hawryluck L, Gold WL, Robinson S, Pogorski S, Galea S, et al. (2004) SARS control and psychological effects of quarantine, Toronto, Canada. Emerg Infect Dis 10: 1206-1212.

271. Hsieh YH, Cheng YS (2006) Real-time forecast of multiphase outbreak. Emerg Infect Dis 12: 122-127.

272. Huang CY, Sun CT, Hsieh JL, Lin H (2004) Simulating SARS: Small-world epidemiological modeling and public health policy assessments. Jasss-The Journal Of Artificial Societies And Social Simulation 7.

273. Hwang DM, Chamberlain DW, Poutanen SM, Low DE, Asa SL, et al. (2005) Pulmonary pathology of severe acute respiratory syndrome in Toronto. Mod Pathol 18: 1-10.

274. Knowles SR, Phillips EJ, Dresser L, Matukas L (2003) Common adverse events associated with the use of ribavirin for severe acute respiratory syndrome in Canada. Clin Infect Dis 37: 1139-1142.

275. Lai EK, Deif H, LaMere EA, Pham DH, Wolff B, et al. (2005) Severe acute respiratory syndrome: quantitative assessment from chest radiographs with clinical and prognostic correlation. AJR Am J Roentgenol 184: 255-263.

276. Lee-Baggley D, DeLongis A, Voorhoeave P, Greenglass E (2004) Coping with the threat of severe acute respiratory syndrome: Role of threat appraisals and coping responses in health behaviors. Asian Journal Of Social Psychology 7: 9-23.

277. Loeb M, McGeer A, Henry B, Ofner M, Rose D, et al. (2004) SARS among critical care nurses, Toronto. Emerg Infect Dis 10: 251-255.

278. Loutfy MR, Blatt LM, Siminovitch KA, Ward S, Wolff B, et al. (2003) Interferon alfacon-1 plus corticosteroids in severe acute respiratory syndrome: a preliminary study. JAMA 290: 3222-3228.

279. MacDonald RD, Henry B, Stuart R (2006) Performance analysis of a medical decision algorithm to mitigate spread of SARS due to interfacility patient transfers. Prehosp Emerg Care 10: 383-389.

280. Maunder RG, Lancee WJ, Balderson KE, Bennett JP, Borgundvaag B, et al. (2006) Long-term psychological and occupational effects of providing hospital healthcare during SARS outbreak. Emerg Infect Dis 12: 1924-1932.

281. Maunder RG, Lancee WJ, Rourke S, Hunter JJ, Goldbloom D, et al. (2004) Factors associated with the psychological impact of severe acute respiratory syndrome on nurses and other hospital workers in Toronto. Psychosom Med 66: 938-942.

282. Mazzulli T, Farcas GA, Poutanen SM, Willey BM, Low DE, et al. (2004) Severe acute respiratory syndrome-associated coronavirus in lung tissue. Emerg Infect Dis 10: 20-24.

283. Muller MP, Richardson SE, McGeer A, Dresser L, Raboud J, et al. (2006) Early diagnosis of SARS: lessons from the Toronto SARS outbreak. Eur J Clin Microbiol Infect Dis 25: 230-237.

284. Muller MP, Tomlinson G, Marrie TJ, Tang P, McGeer A, et al. (2005) Can routine laboratory tests discriminate between severe acute respiratory syndrome and other causes of community-acquired pneumonia? Clin Infect Dis 40: 1079-1086.

285. Nickell LA, Crighton EJ, Tracy CS, Al-Enazy H, Bolaji Y, et al. (2004) Psychosocial effects of SARS on hospital staff: survey of a large tertiary care institution. CMAJ 170: 793-798.

286. Parker MJ, Goldman RD (2006) Paediatric emergency department staff perceptions of infection control measures against severe acute respiratory syndrome. Emerg Med J 23: 349-353.

287. Paul NS, Chung T, Konen E, Roberts HC, Rao TN, et al. (2004) Prognostic significance of the radiographic pattern of disease in patients with severe acute respiratory syndrome. AJR Am J Roentgenol 182: 493-498.

288. Poutanen SM, Low DE, Henry B, Finkelstein S, Rose D, et al. (2003) Identification of severe acute respiratory syndrome in Canada. N Engl J Med 348: 1995-2005.

289. Stinson J, McCartney CJ, Leung A, Katz J (2004) Impact of attending a health care conference in Toronto during the severe acute respiratory syndrome crisis: survey of delegates. Pain Res Manag 9: 137-143.

290. Svoboda T, Henry B, Shulman L, Kennedy E, Rea E, et al. (2004) Public health measures to control the spread of the severe acute respiratory syndrome during the outbreak in Toronto. N Engl J Med 350: 2352-2361.

291. Tang P, Louie M, Richardson SE, Smieja M, Simor AE, et al. (2004) Interpretation of diagnostic laboratory tests for severe acute respiratory syndrome: the Toronto experience. CMAJ 170: 47-54.

292. Tansey CM, Louie M, Loeb M, Gold WL, Muller MP, et al. (2007) One-year outcomes and health care utilization in survivors of severe acute respiratory syndrome. Arch Intern Med 167: 1312-1320.

293. Tolomiczenko GS, Kahan M, Ricci M, Strathern L, Jeney C, et al. (2005) SARS: coping with the impact at a community hospital. J Adv Nurs 50: 101-110.

294. Varia M, Wilson S, Sarwal S, McGeer A, Gournis E, et al. (2003) Investigation of a nosocomial outbreak of severe acute respiratory syndrome (SARS) in Toronto, Canada. CMAJ 169: 285-292.

295. Verbeek PR, McClelland IW, Silverman AC, Burgess RJ (2004) Loss of paramedic availability in an urban emergency medical services system during a severe acute respiratory syndrome outbreak. Acad Emerg Med 11: 973-978.

296. Ward SE, Loutfy MR, Blatt LM, Siminovitch KA, Chen J, et al. (2005) Dynamic changes in clinical features and cytokine/chemokine responses in SARS patients treated with interferon alfacon-1 plus corticosteroids. Antivir Ther 10: 263-275.

297. Webb GF, Blaser MJ, Zhu HP, Ardal S, Wu JH (2004) Critical role of nosocomial transmission in the Toronto SARS outbreak. Math Biosci Eng 1: 1-13.

298. Wilson-Clark SD, Deeks SL, Gournis E, Hay K, Bondy S, et al. (2006) Household transmission of SARS, 2003. CMAJ 175: 1219-1223.

299. Wong T, Wallington T, McDonald LC, Abbas Z, Christian M, et al. (2005) Late recognition of SARS in nosocomial outbreak, Toronto. Emerg Infect Dis 11: 322-325.

300. Zahariadis G, Gooley TA, Ryall P, Hutchinson C, Latchford MI, et al. (2006) Risk of ruling out severe acute respiratory syndrome by ruling in another diagnosis: variable incidence of atypical bacteria coinfection based on diagnostic assays. Can Respir J 13: 17-22.

301. Zhu H, Hu SH, Jona G, Zhu XW, Kreiswirth N, et al. (2006) Severe acute respiratory syndrome diagnostics using a coronavirus protein microarray. Proc Natl Acad Sci U S A 103: 4011-4016.

302. Babyn PS, Chu WC, Tsou IY, Wansaicheong GK, Allen U, et al. (2004) Severe acute respiratory syndrome (SARS): chest radiographic features in children. Pediatr Radiol 34: 47-58.

303. Cheng C, Ng AK (2006) Psychosocial factors predicting SARS-preventive behaviors in four major SARS-affected regions. J Appl Soc Psycho 36: 222-247.

304. Chowell G, Fenimore PW, Castillo-Garsow MA, Castillo-Chavez C (2003) SARS outbreaks in Ontario, Hong Kong and Singapore: the role of diagnosis and isolation as a control mechanism. J Theor Biol 224: 1-8.

305. Cowling BJ, Muller MP, Wong IO, Ho LM, Lo SV, et al. (2006) Clinical prognostic rules for severe acute respiratory syndrome in low- and high-resource settings. Arch Intern Med 166: 1505-1511.

306. Gumel AB, Ruan S, Day T, Watmough J, Brauer F, et al. (2004) Modelling strategies for controlling SARS outbreaks. Proc Biol Sci 271: 2223-2232.

307. Nicholls JM, Butany J, Poon LL, Chan KH, Beh SL, et al. (2006) Time course and cellular localization of SARS-CoV nucleoprotein and RNA in lungs from fatal cases of SARS. PLoS Med 3: e27.

308. Ruan SG, Wang WD, Levin SA (2006) The effect of global travel on the spread of SARS. Math Biosci Eng 3: 205-218.

309. Wong SY, Wong W, Jaakkimainen L, Bondy S, Tsang KK, et al. (2005) Primary care physicians in Hong Kong and Canada--how did their practices differ during the SARS epidemic? Fam Pract 22: 361-366.

310. Wong WC, Wong SY, Lee A, Goggins WB (2007) How to provide an effective primary health care in fighting against severe acute respiratory syndrome: the experiences of two cities. Am J Infect Control 35: 50-55.

311. Yip PSF, Lam KF, Lau EHY, Chau PH, Tsang KW (2005) A comparison study of realtime fatality rates: severe acute respiratory syndrome in Hong Kong, Singapore, Taiwan, Toronto and Beijing, China. Journal Of The Royal Statistical Society Series A-Statistics In Society 168: 233-243.
