# Supplementary material for: Anatomy of the Epidemiological Literature on the 2003 SARS Outbreaks in Hong Kong and Toronto: A Time-Stratified Review
Source: PLoS Med. 2010 May 4;7(5):e1000272. doi: 10.1371/journal.pmed.1000272 (PMC2864302; doi:10.1371/journal.pmed.1000272)
Supplement: Figure S1 — Literature-selection procedure for studies on the SARS epidemic in peer-reviewed journals. (0.12 MB PDF) [file pmed.1000272.s001.pdf]

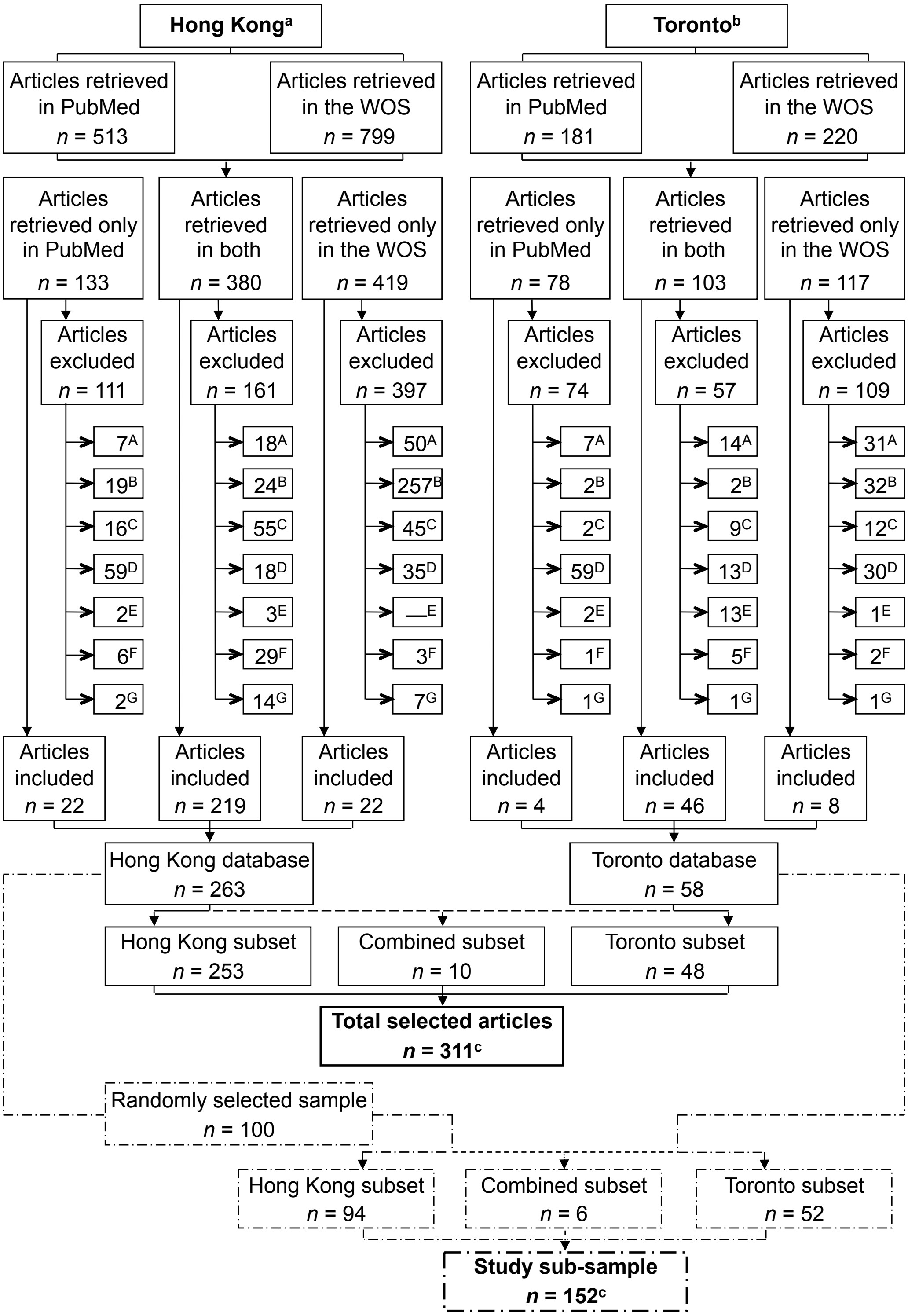

Figure S1. Literature-selection procedure for studies on the SARS epidemic in peer-reviewed journals.

[a]The search equation in PubMed was: (severe acute respiratory syndrome[MAJR] OR SARS) AND "Hong Kong" AND English[LA] AND (Journal article[PT] NOT (review[PT] OR letter[PT] OR

comment[PT] OR editorial[PT] OR news[PT]))-; the search equation in the Web of Science (WOS) was: TS=(severe acute respiratory syndrome OR SARS) AND (TS=(Hong Kong) OR AD=(Hong Kong)) AND Language=(English) AND Document Type=(Article). [b]Search equations identical to those of Hong Kong, except that “Hong Kong” was replaced everywhere by “Toronto”.

The seven following exclusion criteria were applied to identified articles: [A]main study objective was not the study of SARS; [B]data analyzed in the study were not collected in Hong Kong or Toronto; [C]not a study on epidemiology (e.g., animal epizoology); [D]not an original study (e.g., commentary, summary, review); [E]studies using only qualitative methodology; [F]study sample size≤5; [G]at this step, there were 60 remaining articles for which the initial search of the full text of the article was unsuccessful and after sending an email requesting this full text to the corresponding author, we obtained a positive answer in return for 34 articles, resulting in the exclusion of the 26 remaining articles.
